# Supreme-black levels enabled by touchproof microcavity surface texture on anti-backscatter matrix

**DOI:** 10.1126/sciadv.ade4853

**Published:** 2023-01-13

**Authors:** Kuniaki Amemiya, Yuhei Shimizu, Hiroshi Koshikawa, Hiroshi Shitomi, Tetsuya Yamaki

**Affiliations:** ^1^ National Metrology Institute of Japan (NMIJ), National Institute of Advanced Industrial Science and Technology (AIST), Tsukuba 305-8563, Japan.; ^2^ Takasaki Advanced Radiation Research Institute, National Institutes for Quantum Science and Technology (QST), Takasaki 370-1292, Japan.

## Abstract

Emerging immersive high–dynamic range display technologies require not only high peak luminance but also true black levels with hemispherical reflectance below 0.001 (0.1%) to accommodate the wide dynamic range of the human eye (~10^5^). Such low reflectance materials, denoted here as “supreme black,” must exhibit near-perfect surface antireflection, extremely low in-matrix backscattering, and sufficient optical thickness, which, to date, have only been achieved by fragile sparse materials. We demonstrate a record-low hemispherical reflectance below 0.0002 (absorptance above 0.9998) in a touchproof material by satisfying the three requirements with a superwavelength surface microtexture with nanolevel details, low Mie backscattering composition, and optional additional underlayer. Our supreme black finishes are one to two orders of magnitude blacker than previously developed touchproof super-black materials. Thereby, unprecedented black levels enabling an ambient contrast ratio of ≳10^4^ would be provided in display devices, contributing to immersive visual experiences that are critical for seamless remote collaboration and reliable virtual health care.

## Teaser

Superwavelength microtexture with nano details on low Mie backscattering matrix offers touchproof, imperceptibly black finishes.

## INTRODUCTION

**Fig. 1.**
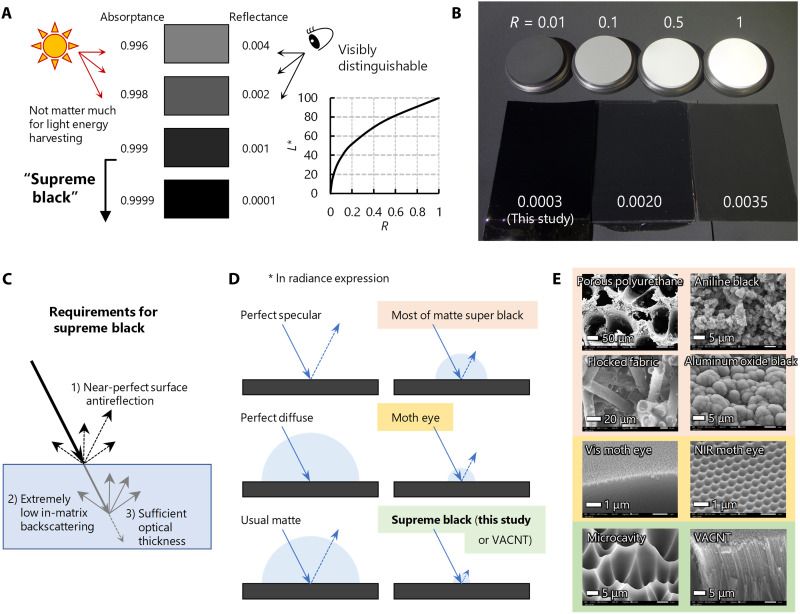
Significance of and requirement for supreme-black levels. (**A**) Significance of supreme-black levels for visual applications. The perceptual lightness L* is not proportional to the (luminous) reflectance *R* of an object; human eyes are much more sensitive to changes in blackness than in whiteness. (**B**) Photograph of super-black materials (lower) and reference standards (upper) with various hemispherical reflectance *R* ranging from 0.0003 to 1. The photo was taken in high–dynamic range mode, where the contrast was compressed to demonstrate the naked-eyesight impression within the limited dynamic range of display devices or printed media (see also fig. S1). (**C**) Three requirements for supreme blackness. (**D**) Schematic diagram showing the forms of surface reflection in terms of radiance representation. (**E**) SEM images of various super-black surfaces.

The human eye perceives blackness based on the amount of reflected light and responds in a manner similar to that described by Weber’s law, according to which the sensation is proportional to the logarithm of the amount of stimulus (*1*). In other words, vision is more sensitive to changes in blackness than in whiteness (Fig. 1A): The naked eye can distinguish among blacks with hemispherical reflectance *R* of 0.004, 0.002, 0.001, or less under sufficient illumination (Fig. 1B). Against such a wide dynamic range of the human eye (five orders of magnitude) (*2*, *3*), the contrast ratios of current standard printed media and display devices are too limited to two to three orders of magnitude (fig. S1). High–dynamic range visual experiences are becoming critical for seamless remote collaboration and reliable virtual health care. Emerging immersive display technologies (*4*–*6*) require not only high peak luminance but also true black levels with hemispherical reflectance *R* of ≪0.001 (denoted here as “supreme black”) to achieve contrast ratios of four to five orders of magnitude even under ambient light illumination. Super-black materials (*7*–*13*), including bioinspired antireflective micro/nanostructures (*14*–*17*), have been attracting a lot of interest. However, most studies have focused on their light energy applications (*8*, *9*, *18*–*20*), which benefit from their thinness (*21*), thermal efficiency (*8*, *9*, *20*, *22*), wavelength selectivity (*19*, *23*–*26*), and adaptability (*27*–*29*), rather than perfect blackness. Previously developed touchproof super-black materials have exhibited hemispherical reflectance of no less than 0.003 to 0.004 (*12*, *30*), which is still too high for use in rigorous light management.

We mapped the requirements for and positioning of supreme black in Fig. 1 (C to E). We considered the following three requirements for achieving an *R* of ≪0.001: (i) almost complete antireflection (AR) at a surface, (ii) extremely low backscattering from the matrix composition, and (iii) sufficient optical thickness. To date, only carbon nanotube (CNT) forests, also called vertically aligned CNTs (VACNTs) (*10*, *31*, *32*), have met all three requirements monolithically: Their sparse growth provides (i) a near-unity effective refractive index, resulting in low Fresnel surface reflection; (ii) isolation of individual CNTs that suppresses Rayleigh backscattering; and (ii) sufficient optical thickness because of the π-band transitions. The early success of VACNT blacks has encouraged research into nanomaterials/nanostructures to achieve extremely low reflectance. However, VACNTs are too fragile for routine use. Biomimetic moth-eye nanostructures certainly contribute to surface index matching (*33*–*35*), while the subwavelength structure (Fig. 1E) leads to some specular reflection (Fig. 1D). Plasmonic metamaterials cancel out reflections by resonance within their negligible thickness compared to the wavelength of interest and can be easily manufactured (*21*, *36*, *37*); however, broadband perfect absorption is inherently difficult. Although a roughened surface reduces some specular reflections, less-designed roughness, such as that of most matte super-black materials (Fig. 1E), increases diffuse reflections (Fig. 1D). Therefore, moth-eye nanostructures are used for low-scattering, nearly perfectly transparent films (*33*, *34*), rather than super-black materials, whereas plasmonic metamaterials are often used as highly absorbing ultrathin films (*21*) and sensors that use resonance shifts (*36*, *37*).

We pursued the development of touchproof supreme-black materials that meet the three requirements shown in Fig. 1C. We had previously successfully fabricated superwavelength microcavity surface texture with nanolevel details on touchproof polymer sheets (Fig. 1E, bottom) that exhibited (i) nearly perfect AR performance (*30*). The resultant blackbody sheet achieved an extremely low reflectance of ~0.0005 in the mid-infrared region (*38*–*40*). Nevertheless, the visible reflectance of a microcavity surface texture on carbon black (CB)–mixed matrix was much higher (>0.003) than expected from the surface AR performance (*30*). Here, we find that a common CB-containing black composition can hardly meet the requirement (ii) shown in Fig. 1C (extremely low in-matrix backscattering).

In this study, we demonstrate a record-low hemispherical reflectance below 0.0002 (absorptance above 0.9998) in a touchproof material by satisfying the above three requirements with the microcavity surface texture, low Mie backscattering matrix, and optional additional underlayer. We identify several black compositions with in-matrix backscattering reflectance of ≪0.001. Cashew nut shell liquid (CNSL) (*41*, *42*)–based black, an unpigmented black urushi lacquer analog, exhibited the lowest backscattering reflectance observed (~0.0001). The resultant touchproof supreme-black finish outperforms most as-grown VACNT blacks (*10*, *31*, *43*) and would provide unprecedented black levels, enabling an ambient contrast ratio of ≳10^4^ in printed media and display devices for high–dynamic range, immersive visual experiences. Despite the necessary thickness of several tens of micrometers, our supreme-black material can still be made in sheet form, making it suitable for practical use.

## RESULTS

**Fig. 2.**
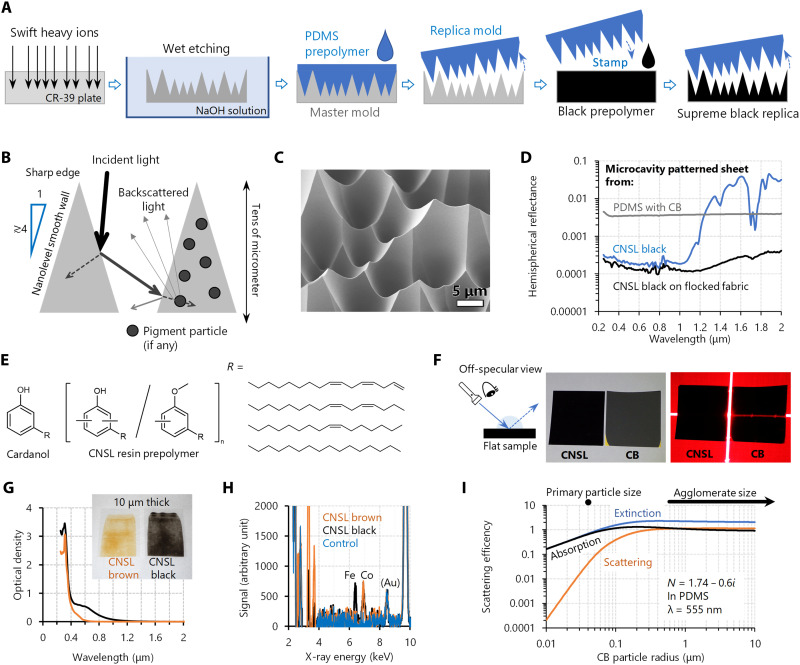
Fabrication, configuration, and characterization of supreme-black finish from anti-backscatter microcavities. (**A**) Fabrication procedure of microcavity-structured supreme-black finish. A microcavity master mold is fabricated by the ion track etching method: A single high-quality conical etch pit is formed per ion track on a CR-39 plastic substrate. Then, the master mold was replicated into a PDMS replica mold, which was then stamped onto a black prepolymer. (**B**) AR principle of microcavity and requirements for achieving ultralow reflectance. It also depicts how light is backscattered by pigment particles, if any. (**C**) SEM image of microcavities on the surface of CNSL-based supreme black. (**D**) UV/Vis/NIR spectral hemispherical reflectance of a CNSL-based supreme-black sheet, compared with the previously reported blackbody sheets (PDMS and CB based) (*30*). (**E**) Molecular structure of CNSL. (**F**) Extremely low light scattering from a flat CNSL-based black film under intense illumination or laser marker, compared with a CB-based one. (**G** and **H**) OD defined as log(1/*T*) (G), where *T* = transmittance of thin CNSL-based brown and black coating (inset), and results of SEM-EDS analysis (H); CNSL black contains iron to form the phenolic complex. (**I**) Simulated Mie scattering efficiency for CB particles with various radii dispersed in PDMS. See also fig. S2 and Materials and Methods for details of the simulation conditions.

Our near-perfect AR microcavity surface texture was fabricated through a combination of ion track etching and replica molding (Fig. 2A) (*30*). Unlike moth eyes, the microcavity size is much larger than the wavelength, resulting in incident light experiencing multiple reflections that enhance the absorption under the geometrical optics approximation (Fig. 2B). To achieve an ultralow reflectance of ≪0.001, the microcavity should have a gradient of ≳4 (*38*, *44*), with nanolevel sharp edges and smooth walls (*30*); otherwise, light confinement partially fails (*45*). Conventional micro- or nanotechnology (*33*–*35*) can hardly fabricate a microcavity template of such quality as is possible with the ion track etching method (*46*) when applied to poly(allyl diglycol carbonate) (PADC; also known as CR-39) substrates (see Materials and Methods for details) (*47*–*49*).

Here, we extended this approach to visibly black materials with various compositions (Fig. 2A). The ion track–based microcavity structure was replicated on the polydimethylsiloxane (PDMS) secondary mold, which was then stamped on the surface of a low-scattering material, namely, CNSL-based black resin (*41*, *42*). CNSL is oxidatively polymerized; thus, the air-permeable PDMS mold allows CNSL to be cured and transfer the microcavity structure with nanolevel details (Fig. 2C). The resulting black sheet exhibited an extremely low hemispherical reflectance of <0.0002 at visible wavelengths (Fig. 2D), which is almost on a par with the world record (*32*), according to reports of appropriate reflectance measurements made using an integrating sphere system. The supreme-black sheet developed in this study is blacker than most as-grown VACNT blacks (*10*, *31*, *43*) and is one to two orders of magnitude blacker than previously developed touchproof super-black materials (*11*, *12*, *20*, *21*, *30*, *50*). Note that the combination of a spectrophotometer and integrating sphere optics, which we also mainly used in this study, is recognized as a standard instrument to evaluate spectral hemispherical reflectance; however, it certainly requires careful use for reliable results (*31*). For validation purposes, we also conducted laser-based hemispherical reflectance measurement with an uncertainty of as small as 0.00002 (= 2 × 10^−5^). See Materials and Methods for detail. Table S1 shows a comparison of laser- and spectrophotometer-based hemispherical reflectance measurements for our two representative CNSL microcavity samples. The results showed good agreement considering the uncertainty for the laser-based measurements (table S2), which supports the reliability of our careful spectrophotometer-based measurements. The averaged reflectance of the CNSL microcavity over the three laser wavelengths (405, 515, and 640 nm) was below 0.0002 even considering the guard band of the uncertainty.

CNSL-based resins (*41*, *42*), which are low-cost and environmentally friendly by-products of cashew nuts, consist of phenolic lipids similar to urushi lacquer (Fig. 2E). Although pristine CNSL is brown, the phenolic vehicle itself turns black as a result of complexing with iron (Fig. 2, F to H) (*51*). The CNSL black is unpigmented; therefore, it has no microparticles to cause Mie backscattering (*52*). The flat-coated glossy CNSL black has almost zero diffuse reflectance (~0.0001), as measured by the specular component–excluded (SCE) method. Even under strong illumination, such as a laser marker, no scattered reflection was observed in the off-specular directions (Fig. 2F), which was not the case for the CB-based flat coating. The impressive deep black in oriental urushi lacquer craftwork similarly stems from this extremely low-scattering property. The microcavity structure on CNSL black resin satisfies both near-perfect surface AR and extremely low in-matrix backscattering to achieve supreme black with almost zero specular and diffuse reflection. (See movies S1 and S2 for a demonstration of the laser pointer disappearing on it.)

We found that a common CB-containing black composition, which we previously tried (*30*), can hardly achieve low in-matrix backscattering by analyzing the reflectance in the infrared region (fig. S2A) and the visible region (Fig. 2D). The light strongly backscattered by the inevitable CB agglomerates (*53*) partly escapes from the microcavity (Fig. 2B and fig. S2B). Such effects cannot be explained by the effective medium approximation (EMA) of the two-component system (*54*) without considering Mie scattering (*52*) from the pigment microparticles. To suppress backscattering by the CB, according to the theoretical calculations (Fig. 2I; see also fig. S2, C to F), the primary nanocarbon particles must be porously deposited without aggregation (*13*, *55*, *56*), although such coatings are fragile.

CNSL black has a low near-infrared (NIR) optical density (OD; Fig. 2G), which can be compensated for by a multilayer structure that satisfies the third supreme-black requirement shown in Fig. 1C (sufficient optical thickness). The lowest reflectance of CNSL supreme black shown in Fig. 2D (indicated by the black line) used an electrostatically flocked nylon pile fabric containing CB as an underlayer to assist in NIR absorption and achieved a reflectance of ≲0.0004. Notably, the NIR reflectance of the pristine flocked fabric is as high as 0.005 to 0.01 (fig. S3). When the pile (Fig. 1E) was embedded in the CNSL black resin, the refractive index was matched, and undesirable scattering at the pile tip was suppressed.

The flexible and breathable PDMS microcavity mold is suitable for use with a variety of target materials for stamping. CNSL needs a couple of days for full oxidative curing. A faster process may be preferable; therefore, we generalized the above multilayer approach to find other possible compositions.

**Fig. 3.**
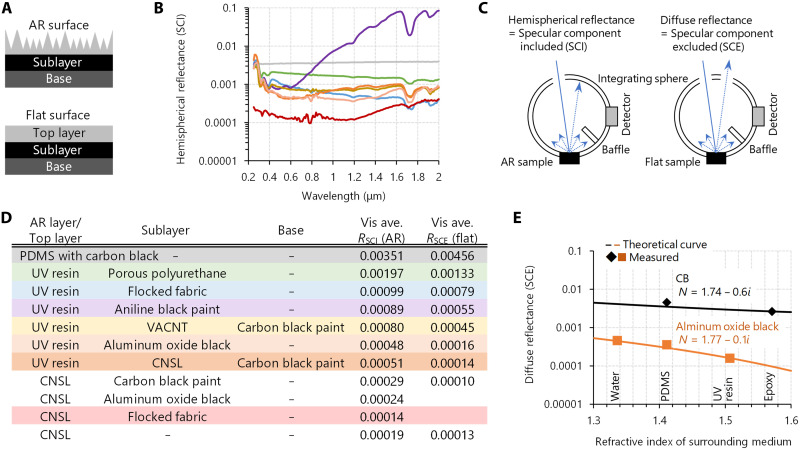
Multilayer variation of supreme-black finishes from anti-backscatter microcavities. (**A**) Schematic illustration of the anti-backscatter multilayer structures. Various existing super-black materials can be chosen as an underlayer. (**B** to **D**) Spectral hemispherical reflectance of microcavity supreme-black finishes fabricated with various combinations of multilayer structures (B), schematic illustration of reflectance measurements under SCI and SCE geometries (C), and average visible hemispherical (SCI) reflectance *R*_SCI_ for the multilayer microcavity supreme-black finishes and average visible diffuse (SCE) reflectance *R*_SCE_ for the corresponding flat samples (D). The same line color in the graph (B) and row color in the table (D) correspond to the same sample. (**E**) Comparison between the measured diffuse (SCE) reflectance *R*_SCE_ and the normalized theoretical reflectance for CB and aluminum oxide black in terms of the dependence on the refractive index of the surrounding medium (see also fig. S4 and Materials and Methods for details).

Ultraviolet (UV)–curable resins permit easy and rapid replication from PDMS microcavity molds. An AR-structured UV-curable resin, which is usually transmissive (and otherwise difficult to cure using UV), requires some light-absorbing underlayer to achieve supreme black (Fig. 3A). The most intuitively suitable underlayer is flat-coated CNSL black, a multilayer structure with which we achieved a visible reflectance of ~0.0005 (Fig. 3B, orange). VACNTs can be used as low-scattering underlayers by embedding them in UV-curable resins, which exhibit ultralow diffuse (SCE) reflectance of ≪0.001 when the surface is flattened (Fig. 3, C and D). Given the microcavity surface texture, VACNT-embedded supreme black exhibited a visible reflectance of ≲0.0008 (Fig. 3B, yellow) without suffering the fragility of as-grown VACNTs. CB-containing flocked fabric can also be embedded in UV-curable resin to form a microcavity surface texture with a NIR reflectance of 0.0004 (Fig. 3B, light blue). At some visible wavelengths, the reflectance slightly exceeds 0.001, which is nevertheless lower than that of all of the following: pristine flocked fabric (fig. S3), a CB-containing porous polyurethane sheet embedded in UV-curable resin with microcavity surface texture (Fig. 3B, green), and our previously developed CB-containing PDMS-based microcavity blackbody sheet (Fig. 3B, gray). Although index matching between the flocked fabric and UV-curable resin suppressed the undesired surface scattering, the reduction of Mie backscattering due to CB content appeared to be limited.

We identified other low-scattering black compositions without CB. Porous aluminum oxide super-black coating, known as Acktar Metal Velvet (*11*), exhibits a visible reflectance of <0.01 (fig. S3). When it was embedded in UV-curable resin with microcavity surface texture, the visible reflectance was <0.0005 (Fig. 3B, peach). This was the blackest sample except for those with CNSL black. The microporous surface of the pristine aluminum oxide black (Fig. 1E), which is not optimal for AR, was index-matched with the UV-curable resin, leading to a reduction in unwanted Mie backscattering. When the aluminum oxide black was embedded in water, PDMS, and UV-curable resin, the diffuse (SCE) reflectance decreased rapidly with increasing refractive index of the medium (Fig. 3E; see also fig. S4, A and B). Unlike CB, black microparticles with a moderate imaginary part of the refractive index *k* can be effectively index-matched when simply embedded in a medium without bothering with nanodispersion, as confirmed by Mie scattering simulation (fig. S4, C and D).

A similar case is that of an acrylic gouache of jet black, a quite black paint containing highly concentrated aniline black (*57*). The as-painted pigment particles (Fig. 1E) exhibit a visible reflectance of 0.015 (fig. S3). When this paint was embedded in UV-curable resin with microcavity surface texture, the visible reflectance was suppressed to <0.0009 (Fig. 3B, purple). Aniline black is nonabsorbent in the infrared region, as is CNSL. However, unlike CNSL, the absorption could not be compensated for by the underlayer (fig. S5A). In the NIR region, the aniline black pigments with too small an imaginary part of the refractive index *k* enhance Mie backscattering, like the “white” pigments (fig. S5, B and C).

Of the various combinations, CNSL black AR on a black flocked fabric exhibited the lowest hemispherical reflectance of ≪0.001, not only in UV, visible (Vis), and NIR but also up to mid-infrared (fig. S6). UV-curable resin AR is slightly more reflective than CNSL black AR (Fig. 3, B and D) but is easier to manufacture while protecting any underlying composition and reducing Mie backscattering. This versatile configuration expands the use of existing super-black materials with application-specific functionalities while enhancing absorptance.

For comparison, our microcavity surface texture, or a commercial moth-eye sheet specialized for the visible or NIR range, was prepared on the aluminum oxide black underlayer, and then the specular component–included (SCI) and SCE hemispherical reflectances were measured using a spectrophotometer (fig. S7A). The diffuse (SCE) reflectance *R*_SCE_ of the moth eye for visible range (height: 200 nm, pitch: 100 nm) was <0.001 in many wavelengths, but the total (SCI) reflectance *R*_SCI_ was approximately 0.001 to 0.005 larger than the *R*_SCE_, corresponding to the specular reflection (fig. S7B). The NIR moth eye (height: 700 nm, pitch: 500 nm) had a similar specular reflection, maintaining *R*_SCI_ ≈ *R*_SCE_ + 0.001. In addition, the *R*_SCE_ exhibited large increases at shorter wavelengths primarily because of the diffraction caused by the periodic array of the NIR moth eye, resulting in an iridescent appearance (fig. S7B). In contrast, our microcavity exhibited reflectance of *R*_SCI_ ≈ *R*_SCE_ < 0.001, suggesting that both specular and diffuse reflection were nearly completely suppressed. Furthermore, UV/Vis/NIR spectrophotometry showed that our microcavity worked over a much wider wavelength range than the moth eyes (fig. S7C). Thus, our superwavelength microcavity with nanolevel details achieves much better AR performance than moth eyes with (sub-)wavelength sizes, as expected from the finite-difference time-domain (FDTD) simulation (*44*).

In addition, we modeled our microcavity surface texture on a matrix including pigment particles (if any) and calculated light propagation by FDTD simulation for a better understanding of the background physics (fig. S8). See Materials and Methods for detailed simulation conditions. The simulation model and calculated hemispherical reflectance are shown in fig. S8, A and B, respectively. For the homogeneous matrix models (CNSL, or the ideally homogeneous PDMS/CB mixture), reflectance values well below 0.00001 were obtained. Thus, microcavity structures fabricated on a homogeneous, scattering center–free matrix would yield extremely low reflectance. The fact that the actual CNSL-based sample exhibited slightly larger reflectance than the calculated values may be due to imperfections in the microcavity fabrication. For PDMS and CB, the matrix/pigment particle model exhibited reflectance of 0.004 to 0.01, which is more than two orders of magnitude larger than the ideally homogeneous model. Furthermore, these reflectance values were several times larger than those of other pigment particle models (aniline black and aluminum oxide black), supporting that the imaginary part of the complex refractive index affects the amount of scattered reflection. The calculated results for the pigment particle models were on the same order of magnitude as, but slightly larger than, the experimental reflectance. The calculation model that is not as rigorous as the actual samples may affect the results (for example, actual pigment particles may have a finer hierarchical structure).

**Fig. 4.**
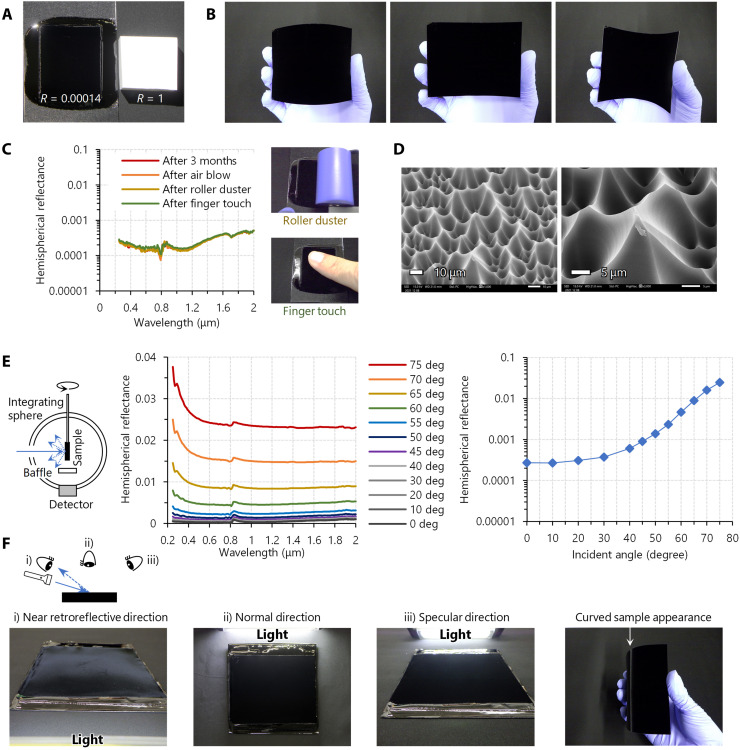
Properties of the supreme-black finish for practical use. (**A**) The contrast ratio of diffusive white against our supreme black is 7.3 × 10^3^ in the best case. (**B**) Flexible CNSL-based supreme-black sheet. (**C**) Durability test of CNSL-based supreme-black sheet. No change was observed in the ultralow reflectance after air blowing, silicone roller duster application, or finger touch. (**D**) SEM image of the CNSL-based supreme-black sheet surface after finger touch. No contamination was observed. (**E**) Incident angle dependence of hemispherical reflectance of the CNSL-based supreme-black sheet. In the rightmost graph, the angle of incidence is plotted on the horizontal axis, and the visible average hemispherical reflectance is plotted on the vertical axis. (**F**) Directional reflection properties of the CNSL-based supreme-black sheet at a large angle incidence. A strong reflection was observed at a certain large incident angle when illuminated from near the viewing direction [(i), see also rightmost photo], whereas there is no strong reflection in the normal (ii) and specular direction (iii).

Our supreme black provides an unprecedented black level toward high–dynamic range visual experiences even under ambient light pollution. The contrast ratio of diffusive white (*R* ≈ 1) against our supreme black (*R* = 0.00014 in the best case) is four orders of magnitude (7.3 × 10^3^; Fig. 4A), which is approaching the human eye’s dynamic range (*2*). In display applications, the ambient contrast ratio ACR (*4*, *6*) is defined as Eq. 1

$$\mathrm{ACR}=\frac{L_{\mathrm{on}}+\frac{I_{\mathrm{amb}}}{\pi }\cdot R_{L}}{L_{\mathrm{off}}+\frac{I_{\mathrm{amb}}}{\pi }\cdot R_{L}}$$ (1)

where *L*_on_ and *L*_off_ are the luminance of on- and off-state pixels, respectively; *I*_amb_ is the ambient light illuminance; and *R*_L_ is the luminous reflectance of a display panel. A state-of-the-art high–dynamic range display (*5*) is capable of *L*_on_ ≈ 2000 cd m^−2^, *L*_on_/*L*_off_ > 10^6^, and *R*_L_ ≈ 0.001 + *R*_b_ (*R*_b_ is luminous reflectance of black matrix covering the nonemitting area), supposing that emission aperture ratio is 0.002 and luminous reflectance of the emitting area is 0.5. By using our supreme-black matrix (*R*_b_ ≈ 0.0002), ACR would be as much as 2.6 × 10^4^, 1.0 × 10^4^, and 50 at *I*_amb_ = 200 lx (living room), 500 lx (office), and 100000 lx (direct sunlight), respectively. In comparison to normal AR coating (*R*_b_ ≈ 0.012) (*4*) and moth eye (*R*_b_ ≈ 0.003; fig. S7A), the ACR would be improved by a factor of >10 and >3, which also means 70 to 90% energy savings for the same ACR. Furthermore, our supreme black has almost no specular reflection component, leading to higher visibility without undesired glare.

Our supreme-black material can be made into a flexible sheet (Fig. 4B). The ultralow reflectance was unchanged after 3 months of storage in a normal environment (Fig. 4C). Spray blowing, roller duster application (under ~5 kPa), or bare finger touch (under ~3 kPa) did not degrade the supreme-black sheet (Fig. 4C and movie S3). The surface microcavities have ridge structures that expand toward their bases and retain the structures even when pressed from above, unlike nanowire-type super-black materials such as VACNTs. In addition, the superwavelength structure of the microcavities prevents them from being buried by hand sebum after the bare finger touch (Fig. 4D), unlike moth-eye nanostructures (fig. S9). Scratching may, however, damage the microcavity structure, depending on the material used. Pencil scratch (6B under 0.75 kg load) or sandpaper linear abrasion (#2000 under ~4 kPa) irreversibly damaged the CNSL AR structure (fig. S10A). Even cellulose cloth abrasion (under ~4 kPa) slightly damaged the CNSL AR structure (fig. S10A); nevertheless, the visible hemispherical reflectance of ≲0.001 was maintained (fig. S10B). Therefore, our supreme-black materials would be touchproof enough to withstand daily use under the possible risk of mild abrasion. In addition, we confirmed that we can restore the performance by pouring additional prepolymer on the damaged surface and reforming the microcavity AR structure.

Given that ambient light enters from the 2π direction, the reflectance against oblique incidence is practically important. Our supreme-black sheet maintained an average visible hemispherical reflectance of <0.001 even at incident angles up to 45° (Fig. 4E). The FDTD simulation (fig. S8C) also supported the incident angle dependence of CNSL-based microcavity reflectance. Lager incident angles result in lower light trapping efficiency as the light approaches perpendicular incidence to the microcavity wall. Although the average visible hemispherical reflectance at 75° incidence of more than 0.02 may not be negligible, the reflected light does not go toward the normal or specular direction but returns to near the incident direction (Fig. 4F). The microcavity at large incident angles works like a retroreflector. Therefore, the black-level performance in the normal direction view is better than that expected from the hemispherical (total) reflectance.

## DISCUSSION

In summary, we developed touchproof supreme-black materials with ultralow visible reflectance of ≪0.001 (<0.0002 in the best case) by achieving almost perfect surface AR, ultralow in-matrix backscattering, and sufficient optical thickness. The ion track–based superwavelength microcavity surface texture with nanodetails was confirmed to exhibit almost perfect AR. We also identified several ultralow Mie backscattering compositions. CNSL, whose phenolic vehicle is black complex with iron, was the lowest-scattering material considered. In addition, other various existing super-black materials embedded simply in transparent resin, without bothering with nanodispersion, demonstrated greatly reduced Mie backscattering because of index matching. The background physics of such low backscattering was also confirmed by FDTD simulations based on optical modeling of the microcavities and pigment particles if any.

Despite the necessary thickness of several tens of micrometers, our microcavity supreme-black material can still be made in sheet form, making it suitable for practical use. Conventional plasmonic metamaterials can be used as highly absorbing ultrathin films of negligible thickness compared to the target wavelength and can be easily manufactured. In contrast, our supreme-black materials exhibit one to two orders of magnitude lower hemispherical reflectance than previously developed touchproof super-black materials including plasmonic metamaterials. The resultant touchproof supreme-black materials would provide unprecedented black levels, enabling an ambient contrast ratio of ≳10^4^ in printed media and display devices for high–dynamic range, immersive visual experiences that are becoming critical toward seamless remote collaboration and reliable virtual health care. A true black matrix in a display device also contributes to substantial energy saving for the sufficient ambient contrast ratio even against modest peak luminance. Our supreme black also prevents stray light to the maximum extent possible, for example, offering high-precision optical measurements as a baseline level reference for spectroscopic analyzers (fig. S11).

Fabrication of the superwavelength microcavity template requires the cyclotron ion beam accelerator. Although the entire process takes days, once a master template is made, the microcavity structure can be easily replicated multiple times. The cyclotron facility that we used in this study currently has a maximum irradiation field of ~100 mm × 100 mm. It will be scaled up by patching multiple PDMS secondary molds (*58*) or final supreme-black sheet products. The flexible and breathable PDMS mold is suitable for use with a variety of target materials for stamping. This versatile fabrication strategy provides design opportunities for supreme-black materials with various application-specific functionalities. Our supreme-black materials would be touchproof enough to withstand daily use under the possible risk of mild abrasion. We anticipate that we will be able to fabricate supreme-black materials with various durable materials other than those discussed here, contributing to a wider range of practical applications under more severe conditions.

## MATERIALS AND METHODS

### Materials

The Baryotrak PADC (also known as CR-39) substrate was purchased from Fukuvi Chemical Industry, Japan. The sodium hydroxide (NaOH) solution for the ion track etching was purchased from FUJIFILM Wako Pure Chemical Corp., Japan. PDMS SIM-360 and curing agent CAT-360, used for replica molds, were purchased from Shin-Etsu Chemical Co. Ltd., Japan. Urashima Urushi Black (Sanko Co. Ltd., Japan), Urushi No. 0112 Black (TOHO Inc., Japan), and Natural Drying Cashew No. 91 Black (Cashew Co. Ltd., Japan) were used as black paints based on CNSL, on which the microcavity structure was stamped. Acrylic UV-curable resins, Star Drop Hard and Star Drop Soft, were purchased from PADICO Co. Ltd., Japan. Two-part epoxy resin consisting of a main resin prepolymer and hardener, Crystal Resin, was purchased from NISSIN RESIN Co. Ltd., Japan.

Moth-eye AR film for the visible range (which is replicated from anodic porous alumina molds; MOSMITE of Mitsubishi Chemical Corporation, Japan) (*33*) was provided by Nakagawa Chemical Inc., Japan. Nickel mold of NIR moth-eye AR structure based on laser interference lithography, HT-NIR-02, was purchased from temicon GmbH, Germany.

Aluminum oxide–based black foil, Metal Velvet, was purchased from Acktar Ltd., Israel. VACNTs of 5 μm long were grown on nickel substrate by Microphase Co. Ltd., Japan. Black nylon electrostatically flocked sheet, Ron-Suede S0.5, was purchased from VEL-SUEDE LTD., Japan. CB-containing porous polyurethane sheet, SuperBlack IR, was purchased from Systems Engineering Inc., Japan. Acrylic Gouache Jet Black and Lamp Black paints were purchased from TURNER COLOUR WORKS LTD., Japan. Aniline black PBk1 pigment, Diamond Black PG143, was purchased from Holbein Artist Materials Inc., Japan. Acetylene CB (50% compressed, average particle size of 42 nm) was purchased from Strem Chemicals Inc., USA.

### Ion beam fabrication of master mold for the light confinement microcavity

CR-39 plastic substrates were cut into ~100 mm × 100 mm pieces. Ion beam irradiation was conducted at the Takasaki Ion Accelerators for Advanced Radiation Application (TIARA) (*59*) of Japan’s National Institutes for Quantum Science and Technology (QST). An ion beam of 200 MeV ^20^Ne^7+^ from an azimuthally varying field cyclotron accelerator was irradiated onto the CR-39 substrates under a vacuum of 10^−3^ Pa in a chamber at the end of a beam port. The beam irradiation density was 1 × 10^6^ to 2 × 10^6^ ions cm^−2^ over the entire area of the substrates. Then, the irradiated CR-39 substrates were etched in 6.4 mol L^-1^ NaOH solution at 70°C until the etch pits (conical microcavities) covered the entire surface of the substrates (for 16 to 18 hours). The etched CR-39 substrates were rinsed in purified water and then dried completely. These whole processes are called the ion track etching method (*46*, *49*). The gradient and depth of the etch pit are determined by the ion species and energy, while the pit opening size is determined by the irradiation density and etching time (*60*). The above irradiation and etching conditions were selected to meet the requirements for microcavity structures with extremely low reflectance (≪0.001) (*30*, *38*, *44*). The microcavity gradient was ≳4, and the microcavity size was greater than the wavelength of interest. The resultant CR-39 substrate was used as a master mold for the following process.

### Fabrication of PDMS replica mold

PDMS prepolymer was prepared by mixing SIM-360 and CAT-360 at a weight ratio of 10:1. The PDMS prepolymer liquid was poured onto the CR-39 master mold and then defoamed in a vacuum desiccator. After curing for a day at room temperature (~22°C), the PDMS replica sheet with the surface microcavities was peeled off from the CR-39 mold. This resultant PDMS replica sheet was used as a secondary mold for the following process.

### Replica molding of the microcavity on various black materials

The AR microcavity surface texture was fabricated on various black materials, with a black underlayer if necessary, in the following manner. A black underlayer coating or a black base sheet was applied or affixed as needed on some substrate [e.g., polyethylene terephthalate (PET) film and aluminum plate]. A CNSL-based black paint, VACNT, or an acrylic gouache black paint was used for the black underlayer coating. An aluminum oxide–based black foil, black nylon electrostatically flocked sheet, or CB-containing porous polyurethane sheet was used for the base black sheet material.

The pigment powder of CB or aniline black was premixed in the epoxy resin main prepolymer using a pestle and mortar. The pigment/prepolymer weight ratio was 5:100 for CB and 2:100 for aniline black. The epoxy resin hardener was then added and mixed at a 2:1 main prepolymer/hardener weight ratio.

A thickness of 150 μm of the CNSL-based black paint, UV-curable resin liquid, or epoxy resin prepolymer containing black pigment was applied on the substrate using a bar coater, and then the PDMS secondary mold was placed on the surface. The sample was then defoamed by pressure applied with a hand roller or using a vacuum desiccator.

In the case of CNSL-based or epoxy resin–based AR structure, after curing for more than a day at room temperature (~22°C), the PDMS secondary mold was peeled off from the cured replica sample. In the case of UV-curable resin-based AR structure, after the UV irradiation (405 and 365 nm, 6 W, for 3 min), the PDMS secondary mold was peeled off from the cured replica sample.

### Preparation of moth-eye black samples for comparison

The NIR moth-eye structure was fabricated on the UV-curable resin by replication from the PDMS secondary mold transferred from the original Ni mold. The UV-curable resin was poured onto the aluminum oxide black (Acktar Metal Velvet) sheet, on which the PDMS secondary mold was placed and defoamed in a vacuum desiccator. After UV irradiation (405 and 365 nm, 6 W, for 3 min), the PDMS mold was peeled off from the cured replica sample.

Moth-eye structures for the visible range of the sheet products (MOSMITE) were used as they are. The UV-curable resin was poured onto the aluminum oxide black (Acktar Metal Velvet) sheet, laminated to the back of the MOSMITE sheet, and UV-cured (405 and 365 nm, 6 W, for 3 min).

### Characterization

#### Reflectance measurement

The spectral reflectance of the samples was measured mainly under the geometric conditions of directional incidence and hemispherical detection of both specular and diffuse components. A spectrophotometer (LAMBDA 1050+, PerkinElmer Inc., USA) with a 150-millimeter-diameter integrating sphere based on porous polytetrafluoroethylene (Spectralon, Labsphere Inc., USA) was used for UV/Vis/NIR reflectance measurement at 8°:di geometry (8° incidence, hemispherical detection: also noted as 8°/*h*), as recommended by the International Commission on Illumination (*61*). The integrating sphere has a 25-millimeter-diameter sample port. Detectors are placed at the bottom of the integrating sphere: a photomultiplier tube for UV/Vis range and an indium gallium arsenide photodiode for NIR range. A Spectralon-based 0.99 (99%) reflectance standard was used as a reference, which was calibrated traceably to the U.S. National Institute of Standards and Technology (NIST). The baseline signal, which could be detected even for an ideal zero-reflectance sample, was determined with the sample port uncovered according to the procedure shown in fig. S11 and subtracted from the acquired sample reflectance data. Notably, the poor performance of the beam stop (nonnegligible amount of reflected light) would cause an overestimation of the baseline amount. We carefully estimated the baseline level by placing our supremely low reflectance sheet on the inner surface of the dark box behind the sample port to prevent the reflected light from returning. A clip-style center-mounted sample holder (PELA9039, PerkinElmer Inc., USA) was used in the integrating sphere to measure the incident angle dependence of the sample reflectance.

Another spectrophotometer (CM-600d, Konica Minolta Japan Inc.) was used to measure the spectral reflectance at the geometry of de:8° (diffuse illumination, 8° viewing angle under SCE conditions). A Fourier transform infrared (FTIR) spectrometer (VERTEX 80v, Bruker Corporation, USA) with a diffusive gold-coated integrating sphere was used for mid-infrared reflectance measurement at 12°:di geometry (12° incidence, hemispherical detection: also noted as 12°/*h*). The Infragold diffusive gold 0.9 (90%) reflectance standard (Labsphere Inc., USA) was used as a reference, which was calibrated traceably to NIST.

#### Validation of reflectance measurement

The combination of a spectrophotometer and integrating sphere optics is recognized as a standard instrument to evaluate spectral hemispherical reflectance; however, it certainly requires careful use for reliable results. For example, the noise level of the detector and the baseline (stray light) level have been found to vary largely by model and year of an instrument. When the reflectance values of the reference standard and the sample differ by many orders of magnitude, we must also pay attention to the linearity of the detector and the throughput variation of the integrating sphere. Detailed notes on the low reflectance measurements can also be found in (*30*, *31*). The uncertainty contributions are from the reflectance standard, the measurement repeatability/reproducibility, the baseline level determination, the instrument linearity, and the integrating sphere throughput. With respect to the UV/Vis/NIR reflectance measurements, throughput variations of the integrating sphere due to sample reflectance were compensated for in double-beam (active reference) mode operation. The integrating sphere of LAMBDA 1050+ has two input ports: One is for the measurement beam and the other is for monitoring the amount of reflected signal at the inner surface of the integrating sphere to correct the throughput, thus canceling the effect of sample exchange on a single measurement port. The relative uncertainty of the 0.99 reflectance standard was 0.5% to 0.9%, depending on the wavelength range. The standard uncertainty due to the measurement repeatability/reproducibility was estimated to be 0.000038 (absolute value). The typical uncertainty due to the baseline level determination with the sample port uncovered was estimated to be 0.000035 (absolute value). The instrument linearity was confirmed by measuring the reflectance of the gray bodies with reflectances of ~0.02 and ~0.10 (Spectralon, Labsphere Inc., USA) by changing the incident light level by two or three orders of magnitude. As a result, the measurement of reflectance levels below 0.001 was possibly overestimated by relatively several percentage points. Considering the noise level in the evaluation, the relative uncertainty due to nonlinearity was conservatively estimated to be 10% to 20%. An offset due to light from the surrounding is negligible because the instrument is completely dark-boxed. The dark level of the spectrophotometer instrument is internally corrected during automated premeasurement calibration. Collectively, the typical total uncertainty for the UV/Vis/NIR measurement was 0.0001 to 0.0002 (95% level of confidence) for a hemispherical reflectance of 0.0002 to 0.001.

For further validation purposes, we also conducted laser-based hemispherical reflectance measurements. A laser-based system improves measurement reliability because it ensures a signal-to-noise ratio with sufficient light intensity and minimizes the influence of stray light due to its high beam directivity. The laser-based system setup in a dark room is shown in fig. S12. Fiber-pigtailed laser diodes were used, with wavelengths of 405, 515, and 640 nm (LP405-SF10, LP520-SF15A, and LP635-SF8, Thorlabs Inc., USA). The laser beam was collimated by a collimator lens and introduced into the 150-millimeter-diameter integrating sphere with Spectraflect (barium sulfate) coating (Labsphere, RT-060-SF) at 8°:di geometry after shaping the beam with an iris and 2-millimeter-diameter pinhole. Steps (i) and (ii) were conducted by switching incident beam ports of the sphere, with the reference and sample always mounted on two 25-millimeter-diameter measurement ports. This allows sample reflectance evaluation by simple comparative measurements since the throughput of the integrating sphere is expected to be maintained. However, because the reflectance values of the reference standard and the sample differ by nearly four orders of magnitude, the nonlinearity of the detector system including the current meter becomes an uncertainty source. Here, a Si-PD (silicon photodiode; Hamamatsu S2386-8K) was used as the detector and a low-noise picoammeter (Keithley 6485) was used as the current meter. Signal levels were around 2 μA and 0.4 nA for the reference and sample reflectance of 0.99 and 0.0002, respectively. Within this range, the response nonlinearity of the Si-PD is less than 1% in relative value, even at a conservative estimate (*62*, *63*). The comparison measurement was repeated five times, and the standard uncertainty due to measurement repeatability was 7.3 × 10^−6^. The measurement reproducibility was checked by swapping the reference and sample mounting ports. The corresponding standard uncertainty was 4.0 × 10^−6^, which may include the slight throughput difference of the integrating sphere for the two measurement ports. Step (iii) determines the baseline signal that would be produced even if a sample with ideally zero reflectance is placed. The measurement beam slightly overfilling the measurement port or slight scattering of the measurement beam in the atmosphere causes the baseline signal. The amount of the baseline signal depends on the shaping of the measurement beam. The good directivity of the laser beam hardly results in overfill, and the baseline signal was less than 1 × 10^−5^ in reflectance equivalent. Because this baseline level was small compared to the reflectance of the sample (~0.0002), it was not subtracted from the sample measurement results, and only considered as an uncertainty (5.5 × 10^−6^ in reflectance equivalent). Step (iv) determines the dark level of the detector, which was negligible (within ±1 × 10^−7^ in reflectance equivalent). Step (v) determines an offset due to light from the surrounding, which was also negligible in a dark room (within ±1 × 10^−7^ in reflectance equivalent). Taking into account the standard uncertainty of the reference standard (0.27% in relative value), the expanded uncertainty of the laser-based hemispherical reflectance measurement was estimated to be as small as 0.00002 (for reflectance < 0.0002, at 95% level of confidence). The uncertainty budgets are summarized in table S2.

The uncertainty analyses of the FTIR measurements are discussed in (*30*). The typical uncertainty at a wavelength of 10 μm was 0.0005 (95% level of confidence) for a hemispherical reflectance of <0.001. The measurements were also validated by comparing the hemispherical reflectance data of the same sample in the overlapping wavelength range (approximately 2 μm) of the UV/Vis/NIR spectrophotometer and the FTIR. As shown in fig. S6, the data obtained with the two spectrometers were consistent at a wavelength of 2 μm, and the measurements were confirmed to be reasonable.

#### OD (transmittance) measurement

A LAMBDA 1050+ spectrophotometer with a Spectralon-based integrating sphere was used for UV/Vis/NIR transmittance measurement. A sample of CNSL thin film coated on a transparent PET sheet (100 μm thick) was placed in front of the beam incident port of the integrating sphere, and its transmittance was measured. An uncoated PET sheet was used as a control sample to determine the net transmittance of the CNSL thin film.

### Scanning electron microscopy

A scanning electron microscope (SEM; JCM-7000 NeoScope, JEOL Ltd., Japan) was used to observe the surface structures of the samples in secondary electron mode. The electron acceleration voltage was 15 kV. The samples were coated with gold to a thickness of ~5 nm using a DC magnetron sputtering apparatus (Vacuum Device Co. Ltd., Japan) before observation to prevent sample surface charging. Energy-dispersive x-ray spectroscopy (EDS) was used to analyze the elemental composition of the CNSL-based paints.

### Photo and video recording

A compact digital camera (G900, RICOH Company Ltd., Japan) was used to record photos and videos of the samples. The photos and the videos were recorded in high–dynamic range mode, except those shown in fig. S1 (C and D), which were recorded in standard dynamic range mode. Figures 2F (rightmost) and 4C and fig. S10A were tone-corrected for ease of viewing.

### Demonstration

A crossed-line laser (Quigo Plus, Robert Bosch GmbH, Germany), a red laser pointer (KPR I, STC Inc., Japan), and a green laser pointer (KPG III, STC Inc., Japan) were used to demonstrate the light annihilation on the supreme-black sample, as shown in Fig. 2F (rightmost) and movies S1 and S2.

### Durability test

A carbon dioxide dust-off spray (KI-1500S, AS ONE Corporation, Japan) and a silicone rubber roller duster (APDCR-30A, AS ONE Corporation, Japan) were used to conduct the durability test of the supreme-black sample, as shown in Fig. 4C and movie S3. Bare finger contact test was conducted under pressure of ~3 kPa. Pencil hardness and linear abrasion tests were also conducted with reference to the recommendations on durability testing of superhydrophobic surface coatings (*64*). A pencil hardness meter (MJ-PHT, Sato Shouji Inc., Japan) was used with a pencil of 6B hardness under 0.75 kg load. Sandpapers (#400 and #2000) and cellulose cloth (BEMCOT M-3II, Ozu Corporation, Japan) were used for linear abrasion test at a single cycle under the pressure of ~4 kPa by using 0.25kg load on the area of 25 mm × 25 mm.

### Mie scattering simulation and analysis

Mie scattering simulations were conducted using the MiePlot software provided by Laven (*65*, *66*). The scattering efficiency *Q* = *C*/π*r*^2^, albedo *a* = *Q*_sca_/*Q*_ext_, scattering phase function *p*(θ), and asymmetry factor *g* = *∫ p*(θ) cosθ *d*Ω/*∫ p*(θ) *d*Ω were calculated for each pigment particle with a complex refractive index *N* = *n* – *ik* and surrounding medium with a refractive index *n*_med_, where *C* is the scattering cross section, *r* is the particle radius, and θ is the scattering angle. *Q*_ext_ is the sum of *Q*_sca_ and *Q*_abs_, where *Q*_ext_, *Q*_sca_, and *Q*_abs_ are the scattering efficiencies for total extinction, scattering, and absorption, respectively.

Simulations were conducted mainly at a wavelength λ = 555 nm, at which the human eye’s sensitivity is at its maximum. To characterize the aniline black pigment in the NIR wavelength range, simulations were conducted at λ = 1.5 μm. It was assumed that the incident light was unpolarized and that the particle radius followed a normal distribution with 20% dispersion. As the complex refractive indices of the pigment particles, *N* = 1.737 − 0.6*i* for CB, *N* = 1.770 − 0.1*i* for aluminum oxide black, and *N* = 1.852 − *ik*, *k* = 0 to 0.5, for aniline black were used. The values of *n* and *k* for CB and *n* for aluminum oxide black and aniline black were taken from the literature (*67*–*69*). Values of *n*_med_ = 1 for air, 1.336 for water, 1.411 for PDMS, 1.507 for UV-curable resin, and 1.571 for epoxy resin were used as the refractive indices of the surrounding media. These values were derived by the Fresnel equation from the specular reflectance data of the flat-finished media.

Reflectance due to the backscattering by pigment particles is determined by the asymmetry factor and the albedo and is independent of particle concentration in a semi-infinite slab (*13*, *70*). The larger the asymmetry factor is and the smaller the albedo is, the lower the backscattered reflectance is. Because of the complexity of the Mie scattering equation, it is difficult to express its dependence on the refractive index of a pigment particle and surrounding medium in a simple equation. Nevertheless, the amount of reflection at the pigment microparticle surface is expected to follow the Fresnel equation; the qualitative trend of backscattered reflectance can be explained with respect to the refractive index of the pigment particle *N* and the surrounding medium *n*_med_ as follows

$$R_{\mathrm{SCE}}\propto {|\frac{N-n_{\mathrm{med}}}{N+n_{\mathrm{med}}}|}^{2}$$

where *R*_SCE_ is the diffuse reflectance of the sample for the SCE geometry. In Fig. 3E and fig. S4B, the theoretical curve is plotted for the values of the above Fresnel equation, normalized by the experimental data at *n*_med_ = 1.507 for the aluminum oxide black and at *n*_med_ = 1.571 for CB.

### FDTD simulation

The free and open-source software package MEEP (*71*) from Massachusetts Institute of Technology, USA was used for FDTD simulation. We assumed a simplified model of microcavities and pigments, and calculated the hemispherical reflectance for the representative compositions of the actual samples in this study. As shown in fig. S8A, we constructed a model in which spherical pigment particles with diameters of 1 or 2 μm are placed in a matrix of inverted conical microcavity structures with opening diameters of 2 or 4 μm and aspect ratios of 5. The volume filling factor of the pigment particles is approximately 4.4%, which is comparable to our actual pigment-containing samples. We assumed a periodic boundary condition with this unit cell in the in-plane direction. A perfectly matched layer was set just below the pigment particles to prevent light traveling deeper than them from returning. We believe that this simplified modeling is a reasonable assumption, since the pigment particles in the topmost layer have the greatest influence on backscattering, and even if there is a return of light from the deeper site, it is expected to be enough attenuated by this pigment layer. Although the size of the microcavity model is smaller than the actual sample, it is still sufficiently larger than the target wavelength (~555 nm) to expect the same degree of surface AR effect. The parameters of the complex refractive indices of the matrix and pigment particles are summarized in the table in fig. S8B. Some calculations were also conducted for homogeneous models without pigment particles: CNSL and an ideally homogeneous PDMS/CB mixture were assumed. The complex refractive index for CNSL was determined from the specular reflectance data of a flat-finished sample and the OD data shown in Fig. 2G. For the ideally homogeneous PDMS/CB mixture, the complex refractive index was derived with the Maxwell-Garnett approximation, a kind of EMA (*54*).

## Supplementary Materials

### This PDF file includes:

Figs. S1 to S12 Tables S1 and S2 References
