# Supplementary material for: Supreme-black levels enabled by touchproof microcavity surface texture on anti-backscatter matrix
Source: Sci Adv. 2023 Jan 13;9(2):eade4853. doi: 10.1126/sciadv.ade4853 (PMC9839331; doi:10.1126/sciadv.ade4853)
Supplement: Supplementary file 1 — Figs. S1 to S12 Tables S1 and S2 References [file sciadv.ade4853_sm.pdf]

Science Advances
AAAS

Supplementary Materials for

Supreme-black levels enabled by touchproof microcavity surface texture on anti-backscatter matrix

Kuniaki Amemiya *et al.*

Corresponding author: Kuniaki Amemiya, k.amemiya@aist.go.jp

Sci. Adv. **9**, eade4853 (2023)
DOI: 10.1126/sciadv.ade4853

The PDF file includes:

Figs. S1 to S12
Tables S1 and S2
Legends for movies S1 to S3
References

Other Supplementary Material for this manuscript includes the following:

Movies S1 to S3

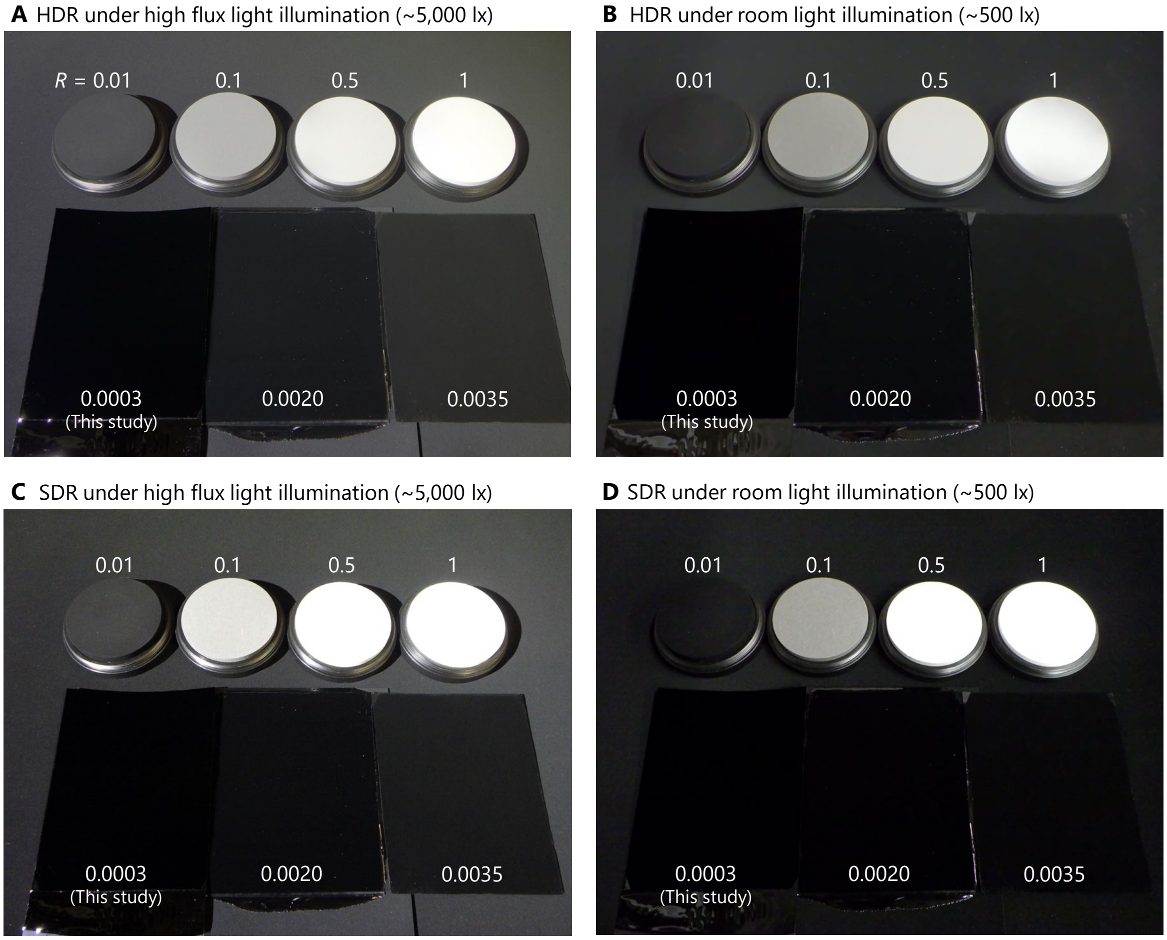

Fig. S1.

Demonstration of the bright/dark dynamic range. (**A** to **D**) Super-black materials (lower) and reference standards (upper) with various hemispherical reflectance R from 0.0003 to 1 photographed under strong light or room light illumination in (A and B) high dynamic range (HDR), and (C and D) standard dynamic range (SDR) modes of a compact digital camera. Human eyes, image sensors, display devices, and printed media have typical dynamic ranges of 5, 4, 3, and 2 orders of magnitude, respectively. Therefore, SDR mode can hardly display the naked-eyesight impression, resulting in whiteout under bright lighting (C) and blackout under dark lighting (D). HDR photography pseudo-expands the dynamic range by combining multiple images with different exposures and compresses the contrast to match the dynamic range of the display devices or printed media, expressing the perceptual lightness differences regardless of the lighting (A and B). Of course, the details are still different from the actual naked-eyesight impression (at the mid-level reflectance, for example).

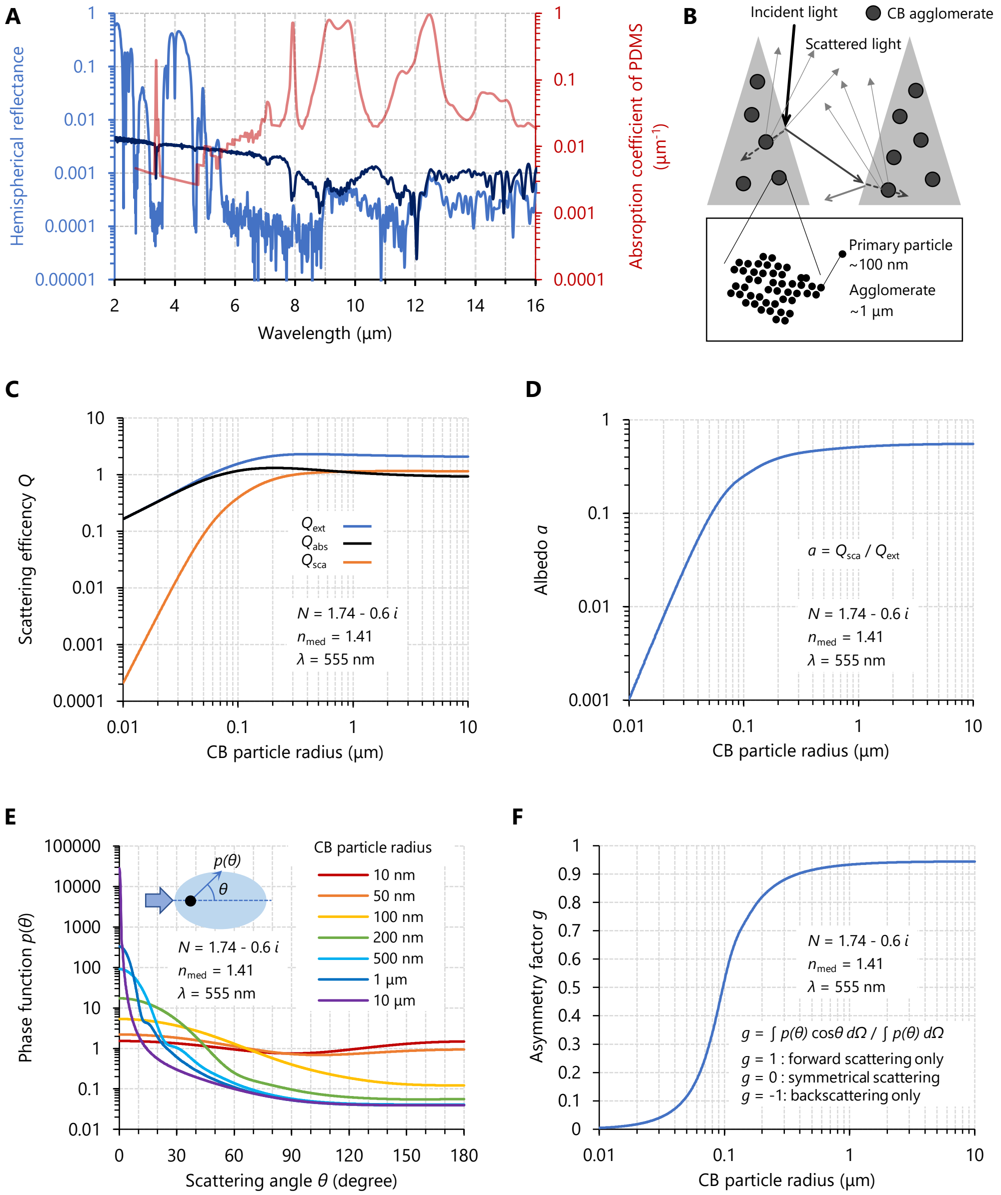

Fig. S2.

Backscattering from a carbon black particle. (**A**) Infrared hemispherical reflectance of PDMS-based microcavity blackbody sheets (*30*) with (dark blue line) and without (light blue line) carbon black (CB) pigments. The red line is the spectral absorption coefficient of PDMS derived from the data in Ref. (*72*). At some wavelengths for which the PDMS showed weak infrared absorption, the CB-containing blackbody sheet exhibited higher reflectance than the CB-less sheet. (**B**) Schematic illustration of how the light scattered by CB agglomerates (*53*) escapes from the microcavity structure. (**C** to **F**) Simulated Mie scattering efficiency (C), albedo (D), scattering phase function (E), and asymmetry factor (F) for CB particles with various radii dispersed in PDMS. See Materials and Methods section for details of the simulation conditions. Reflectance due to backscattering by pigment particles is determined by the asymmetry factor and the albedo and is independent of the particle concentration in a semi-infinite slab (*13*, *70*). The larger the asymmetry factor is and the smaller the albedo is, the lower the backscattered reflectance is. In the case of CB, backscattering is only sufficiently reduced if the primary nanoparticles are highly dispersed in the matrix without aggregation.

A

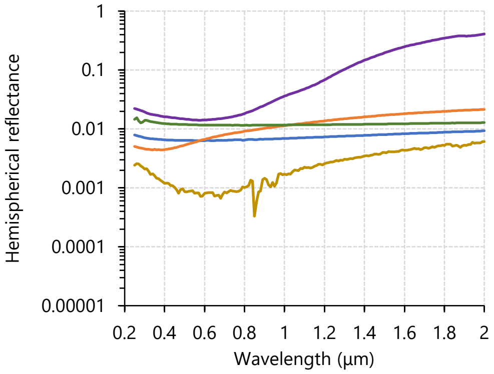

B

Material	Vis ave. R_{SCI}
Aniline black paint	0.01482
Porous polyurethane	0.01185
Flocked fabric	0.00640
Aluminum oxide black	0.00605
VACNT	0.00088

C

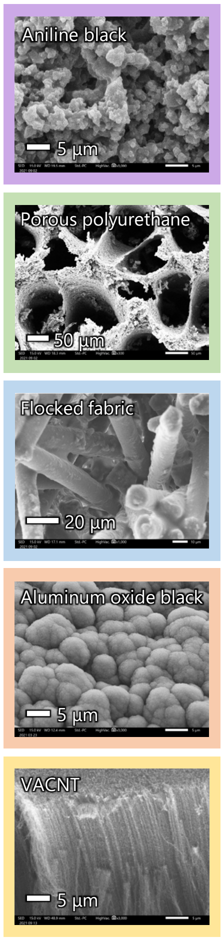

Fig. S3.

Properties of various existing super-black materials. (**A** to **C**) UV/Vis/NIR spectral hemispherical reflectance (A), visible average hemispherical reflectance R_{SCI} (B), and surface SEM images (C) of various existing super-black materials. The same line color in (A), row color in (B), and frame color in (C) correspond to the same sample.

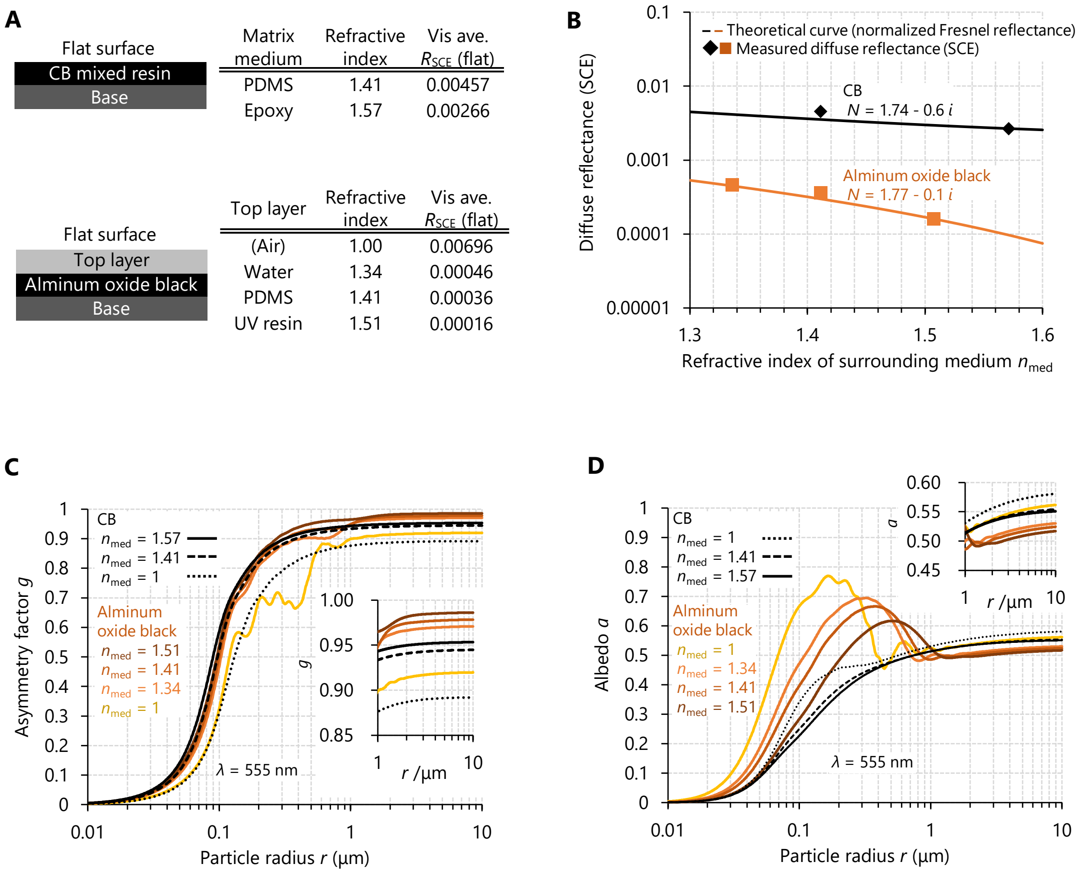

Fig. S4.

Dependence of Mie scattering on the refractive index of surrounding media. (**A**) Schematic illustration of the examined compositions: One is the CB mixed resin. The other is the multi-layer structure with aluminum oxide black as the underlayer and uncoated, water, PDMS, or UV-curable resin as the top layer. The average visible diffuse (SCE) reflectance R_{SCE} for each composition is summarized in the tables. (**B**) Comparison between the measured diffuse reflectance R_{SCE} and the normalized theoretical Fresnel reflectance in terms of the dependence on the refractive index of the surrounding medium (see Materials and Methods section for details). (**C** and **D**) Simulated Mie scattering asymmetry factor (C), and albedo (D) for CB and aluminum oxide black particles with various radii dispersed or embedded in various media. See Materials and Methods section for detailed simulation conditions. The larger the asymmetry factor is and the smaller the albedo is, the lower the backscattered reflectance is. Considering here the actual pigment particles are on the order of microns in size, index matching with the surrounding medium can increase the asymmetry factor and reduce the albedo. Notably, the relatively large imaginary part of refractive index *k* of the pigment particles, as in CB, limits the effect of index matching with the medium, whereas the moderate *k*, as in aluminum oxide black, allows more effective index matching with the medium.

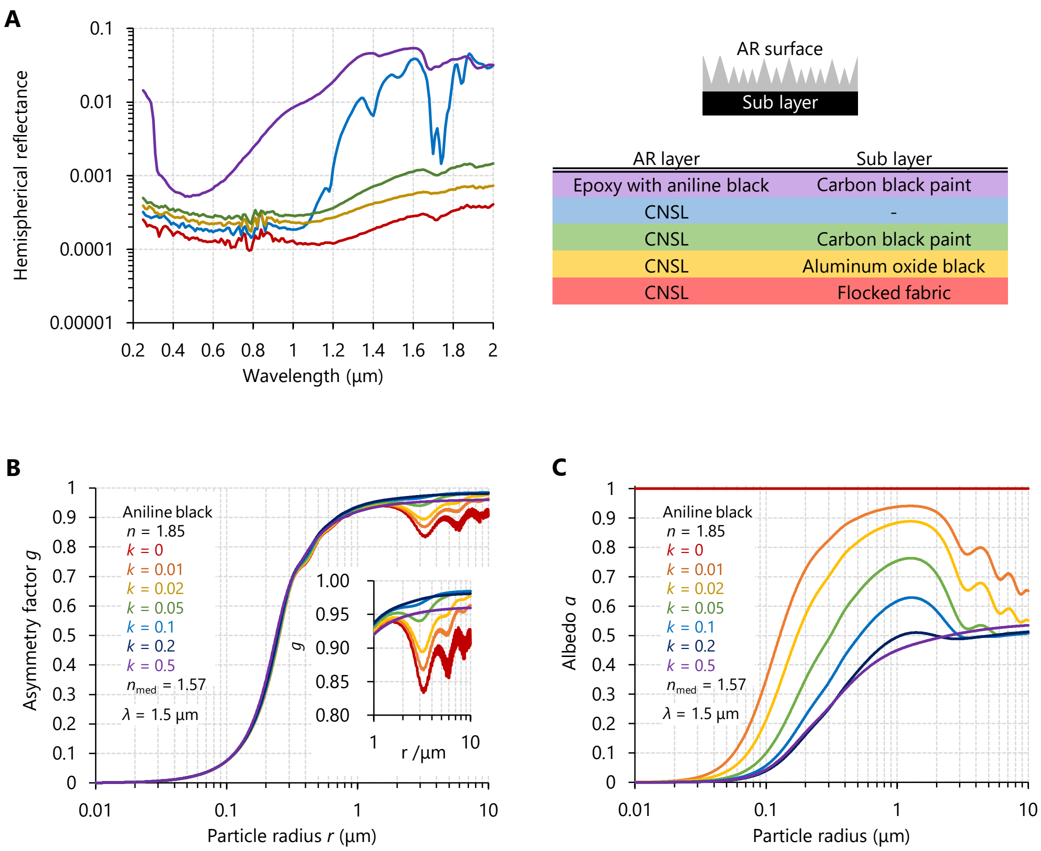

Fig. S5.

Near-infrared reflectance of the multi-layered microcavity supreme blacks. (**A**) Both CNSL black resin and aniline-black-containing epoxy had poor near-infrared absorption and large hemispherical reflectance. In the case of CNSL black, the underlayers (CB-containing compositions or aluminum oxide black) compensated near-infrared absorption and suppressed hemispherical reflectance. However, this was not the case for aniline-black-containing epoxy. (**B** and **C**) Simulated Mie scattering asymmetry factor (B) and albedo (C) for aniline black particles with various radii dispersed in epoxy resin. See Materials and Methods section for detailed simulation conditions. The smaller the asymmetry factor is and the larger the albedo is, the higher the backscattered reflectance is. Aniline black behaves like a “white” pigment in the near-infrared range where the imaginary part of refractive index k is quite small.

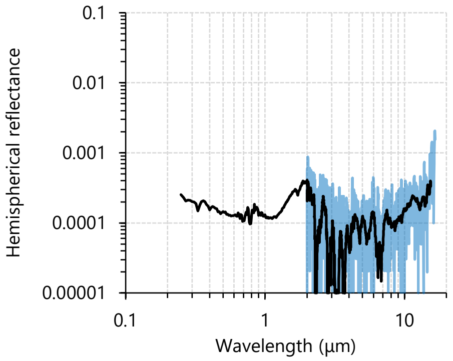

Fig. S6.

Broadband spectral hemispherical reflectance of microcavity supreme-black sheet. The CNSL-based microcavity supreme-black finish on the flocked fabric underlayer exhibited a hemispherical reflectance of well below 0.001 over the ultra-wideband of UV/Vis/NIR/MIR. The UV/Vis/NIR spectrum was measured by a spectrophotometer, and the mid-infrared (MIR) spectrum was measured by an FTIR spectrometer. The black line in the MIR spectrum represents the moving average of the neighboring 20 acquired data points (blue line).

A

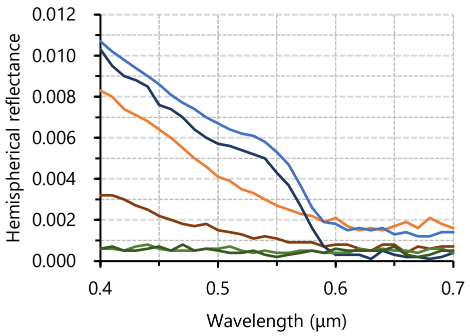

AR layer	Sub layer	Vis ave. R
Vis moth eye SCI	Aluminum oxide black	0.00366
Vis moth eye SCE	Aluminum oxide black	0.00137
NIR moth eye SCI	Aluminum oxide black	0.00494
NIR moth eye SCE	Aluminum oxide black	0.00398
UV resin microcavity SCI	Aluminum oxide black	0.00053
UV resin microcavity SCE	Aluminum oxide black	0.00048

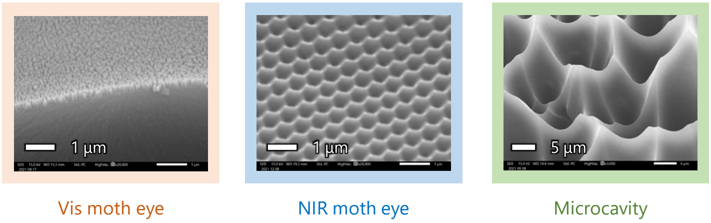

B

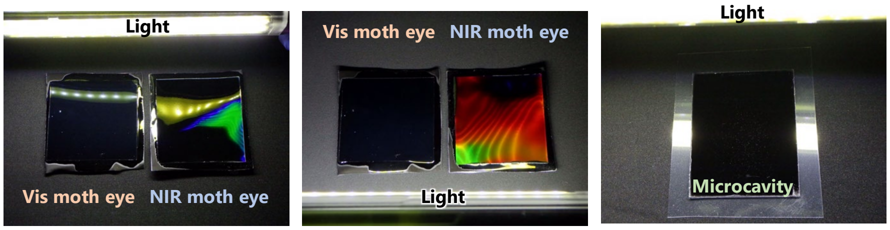

C

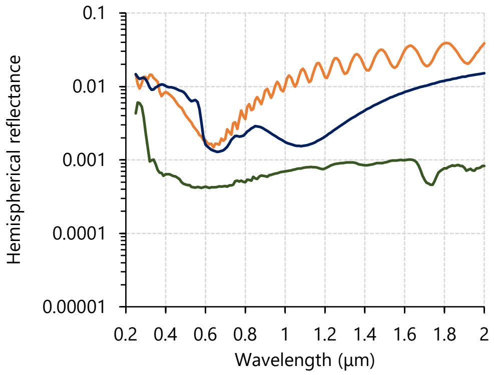

Fig. S7.

Comparison between microcavity supreme black and moth-eye black. (**A**) Specular component-included (SCI) or -excluded (SCE) spectral hemispherical reflectance of the samples in the visible range (graph). The configurations of the samples are summarized in the table. The same line color in the graph, row color in the table, and frame color in the surface SEM image correspond to the same sample. (**B**) Reflection appearance of the moth eye and microcavity samples under the linear LED array illumination. The two moth-eye samples exhibited obvious specular reflection. The NIR moth-eye sample had an iridescent coloration due to diffraction effects. Neither specular reflection nor coloration was observed on the microcavity sample. (**C**) UV/Vis/NIR spectral hemispherical reflectance (SCI) of the moth eyes (orange and blue lines) and the microcavity (green line) samples. The two moth-eye samples exhibited low reflectance of ~0.002 only in a narrow wavelength range, whereas the microcavity sample showed ultralow reflectance of < 0.001 over the much wider wavelength range.

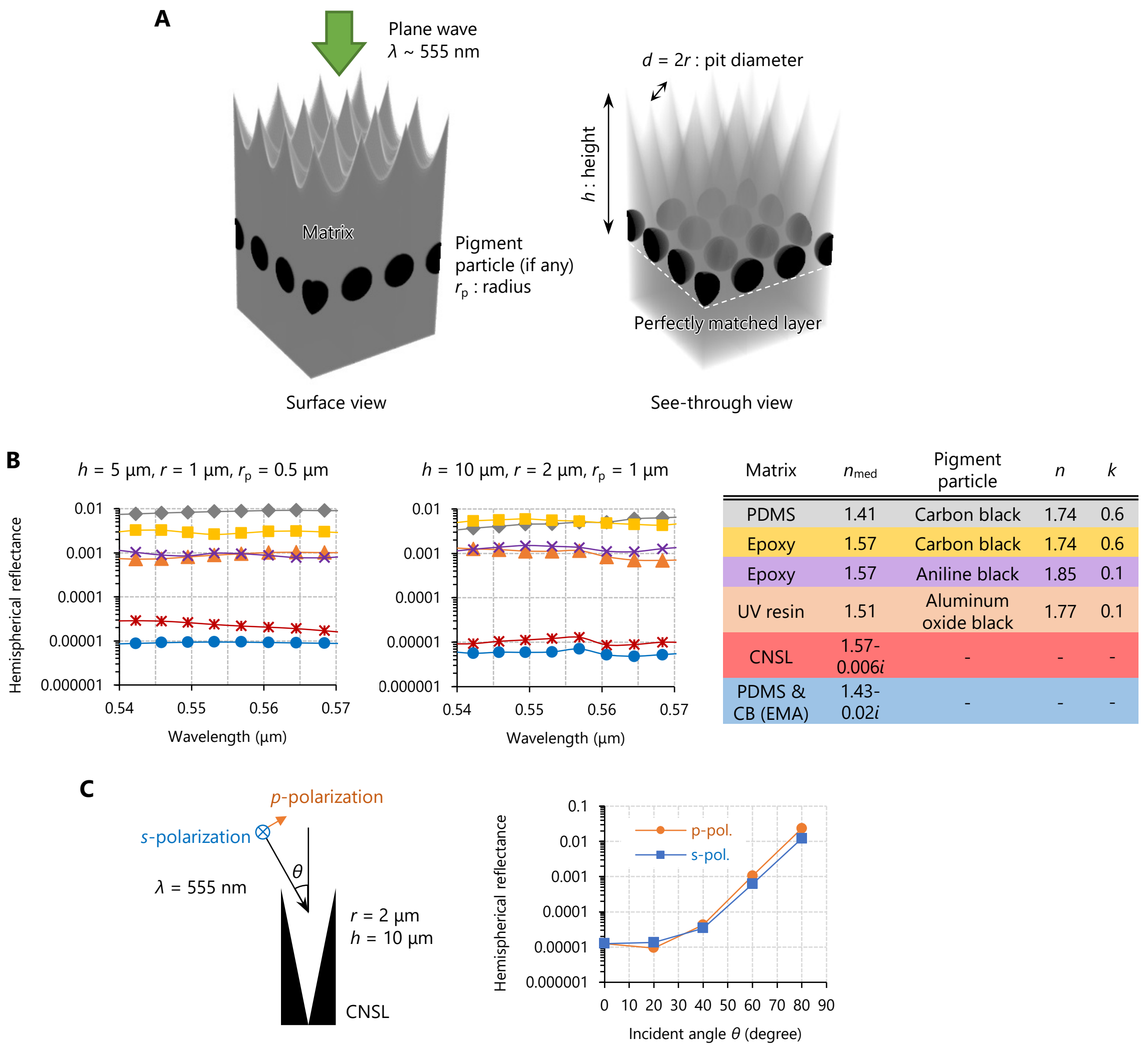

Matrix	n_{med}	Pigment particle	n	k
PDMS	1.41	Carbon black	1.74	0.6
Epoxy	1.57	Carbon black	1.74	0.6
Epoxy	1.57	Aniline black	1.85	0.1
UV resin	1.51	Aluminum oxide black	1.77	0.1
CNSL	1.57-0.006i	-	-	-
PDMS & CB (EMA)	1.43-0.02i	-	-	-

Fig. S8.

Finite-difference time-domain (FDTD) simulation of the hemispherical reflectance of microcavity (supreme-)black materials. (**A**) Simulation model of the microcavity (supreme-)black materials. Pigment particles (if any) were placed around the bottom of conical pits of microcavities. (**B**) Simulated hemispherical reflectance for the microcavity (supreme-)black material models. The table summarizes the simulation conditions of compositions. The same line color in graphs and row color in the table correspond to the same condition. (**C**) Simulation model for the oblique incidence (left) and the simulated hemispherical reflectance depending on the incident angle. See Materials and Methods section for detailed simulation conditions.

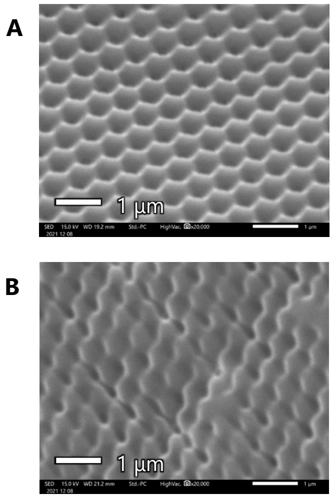

Fig. S9.

S**urface SEM image of a moth eye before and after finger touching.** (**A**) Before finger touching, (**B**) after finger touching. Unlike the superwavelength microcavity (Fig. 4D), the moth-eye surface texture of submicron size was buried by hand sebum after touching.

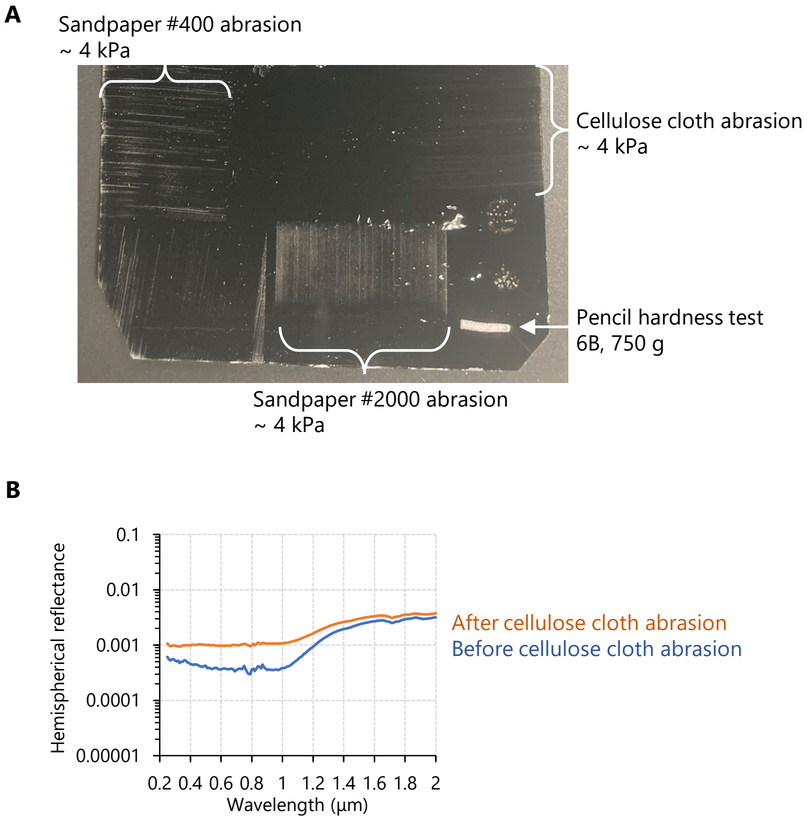

Fig. S10.

Additional durability test of a CNSL-based microcavity supreme-black sheet. (**A**) The results of the pencil hardness test and single-cycle linear abrasion test. This photo was tone-corrected for ease of viewing. See Materials and Methods section for detailed test conditions. (**B**) The spectral hemispherical reflectance of the CNSL-based microcavity supreme-black sheet before and after cellulose cloth abrasion under ~4 kPa. The ultralow reflectance of $\lesssim 0.001$ was still maintained after the abrasion.

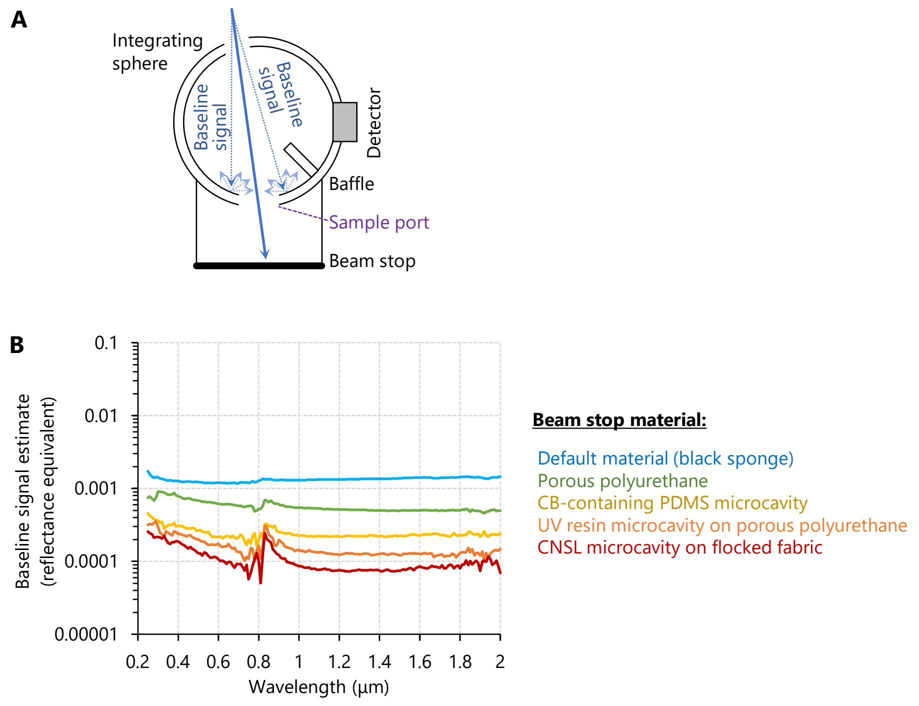

Fig. S11.

Application of the supreme-black finish to stray light elimination. (**A**) Schematic diagram of the integrating sphere optics for spectral hemispherical reflectance measurement. A dark box that also serves as a beam stop is placed behind the sample port. If a zero-reflectance sample is placed, a zero signal should be detected. In reality, slightly imperfect collimation of the incident beam causes the light scattered around the sample port to be detected as a non-zero baseline signal. The baseline level must be subtracted from the signal of the sample measurement. (**B**) Baseline signal estimation with various beam-stop materials. If the reflectance of the beam stop is not sufficiently low, a baseline level would be overestimated. The beam stop from the CNSL-based supreme-black sheet with the black flocked fabric underlayer reduced the returned light into the integrating sphere negligibly, and the accurate baseline level was estimated. All the spectral hemispherical reflectance values in this study were corrected by subtracting this baseline level.

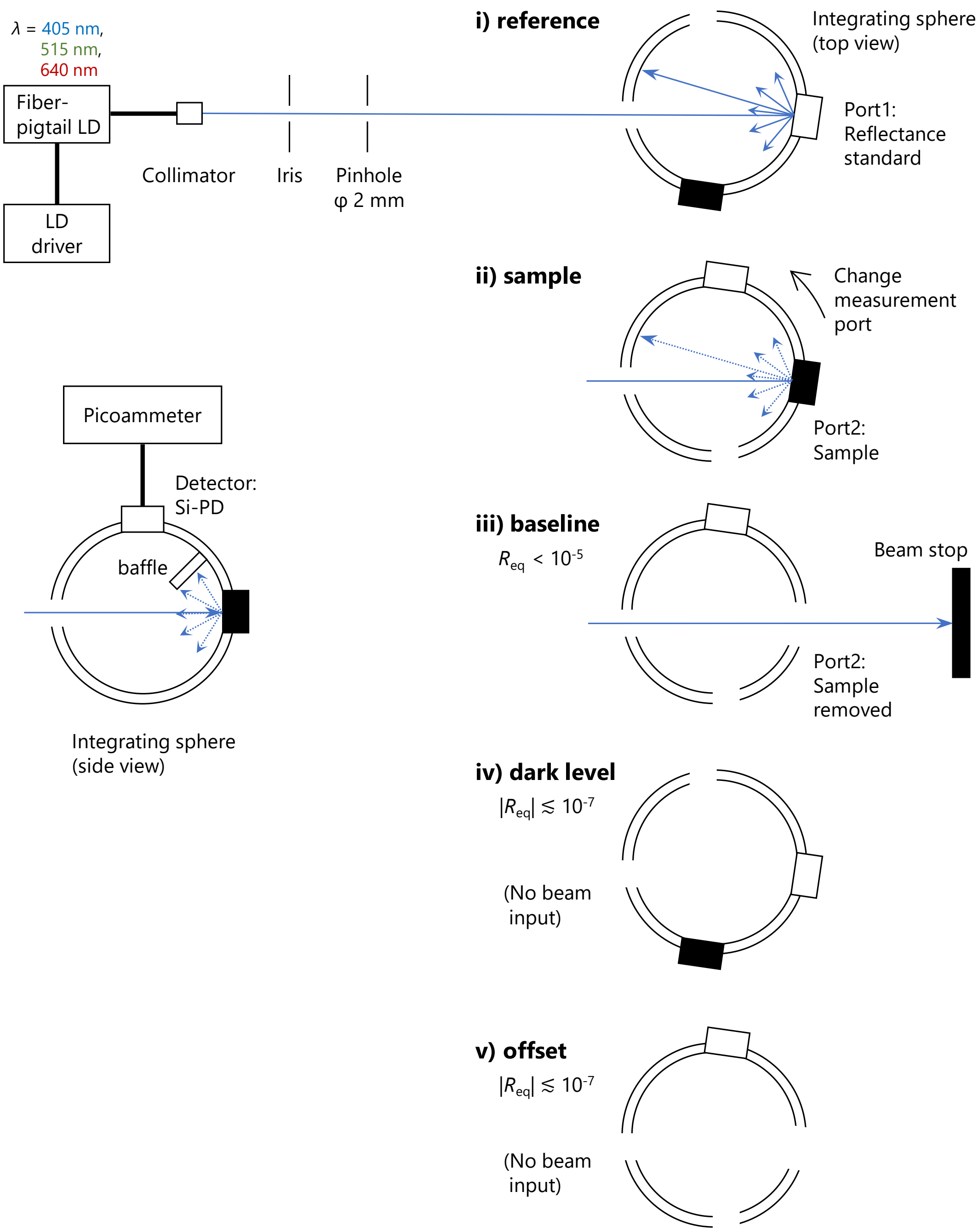

Fig. S12.

Procedures for laser-based hemispherical reflectance measurements. ii) sample signal was divided by i) reference signal and multiplied by the calibrated reference reflectance to determine the hemispherical reflectance of the sample. iii) baseline signal was not subtracted from the ii) sample signal and only considered as an uncertainty source because its value ($< 10^{-5}$ hemispherical reflectance equivalent R_{eq}) was small compared to the sample reflectance (~0.0002) when using the well-collimated laser. iv) dark level of the detector and v) offset due to light from the surrounding were measured to correct the iii) baseline signal estimation but were negligible when measured in a dark room. See Materials and Methods section for detailed procedures.

Table S1.
Comparison of laser- and spectrophotometer-based hemispherical reflectance measurements.

Wavelength (nm)	CNSL microcavity[a)]		CNSL microcavity on flocked fabric[b)]	
	Laser-based setup	Spectro-photometer	Laser-based setup	Spectro-photometer
640	0.000166 ± 0.000020	0.000174	0.000169 ± 0.000020	0.000184
515	0.000166 ± 0.000020	0.000185	0.000190 ± 0.000020	0.000193
405	0.000187 ± 0.000020	0.000219	0.000198 ± 0.000020	0.000230

[a)]A sample fabricated for this comparison only.
[b)]The same sample for which the bare finger contact test was conducted (the results were shown in Fig. 4C). This comparison was conducted after another year.

Table S2.
Uncertainty budget of laser-based hemispherical reflectance measurements.

Component	Standard uncertainty	Type	
Reference reflectance standard	0.0027	B	Relative
Detector nonlinearity	0.0058	B	Relative
Baseline	0.0000055	B	Absolute
Measurement port reproducibility	0.0000040	B	Absolute
Measurement repeatability	0.0000073	A	Absolute
Combined standard uncertainty	0.000010[a)]		Absolute
Expanded uncertainty (95 % level of confidence)	0.000020[a)]		Absolute

[a)]In the case of hemispherical reflectance $\lesssim 0.0002$

Movie S1.
A red-line laser (~0.7 mW) was illuminated on the microcavity black sheets based on CNSL black (left) and CB-containing PDMS (right).

Movie S2.
A green-line laser (~0.7 mW) was illuminated on the microcavity black sheets based on CNSL black (left) and CB-containing PDMS (right).

Movie S3.
Durability tests (blower, roller duster, and finger touch) were conducted on the microcavity supreme black based on CNSL.

REFERENCES AND NOTES

1. A. E. O. Munsell, L. L. Sloan, I. H. Godlove, Neutral value scales. I. Munsell neutral value scale. *J. Opt. Soc. Am.* **23**, 394–411 (1933).

2. H. Seetzen, W. Heidrich, W. Stuerzlinger, G. Ward, L. Whitehead, M. Trentacoste, A. Ghosh, A. Vorozcovs, High dynamic range display systems. *ACM Trans. Graph.* **23**, 760–768 (2004).

3. G. Tan, Y. Huang, M.-C. Li, S.-L. Lee, S.-T. Wu, High dynamic range liquid crystal displays with a mini-LED backlight. *Opt. Express* **26**, 16572–16584 (2018).

4. E. Hsiang, Z. He, Y. Huang, F. Gou, Y. Lan, S. Wu, Improving the power efficiency of micro-LED displays with optimized LED chip sizes. *Crystals* **10**, 494 (2020).

5. G. Biwa, A. Aoyagi, M. Doi, K. Tomoda, A. Yasuda, H. Kadota, Technologies for the crystal LED display system. *J. Soc. Inf. Disp.* **29**, 435–445 (2021).

6. E. Hsiang, Z. Yang, Q. Yang, Y. Lan, S. Wu, Prospects and challenges of mini-LED, OLED, and micro-LED displays. *J. Soc. Inf. Disp.* **29**, 446–465 (2021).

7. Y.-F. Huang, S. Chattopadhyay, Y.-J. Jen, C.-Y. Peng, T.-A. Liu, Y.-K. Hsu, C.-L. Pan, H.-C. Lo, C.-H. Hsu, Y.-H. Chang, C.-S. Lee, K.-H. Chen, L.-C. Chen, Improved broadband and quasi-omnidirectional anti-reflection properties with biomimetic silicon nanostructures. *Nat. Nanotechnol.* **2**, 770–774 (2007).

8. L. Zhou, Y. Tan, J. Wang, W. Xu, Y. Yuan, W. Cai, S. Zhu, J. Zhu, 3D self-assembly of aluminium nanoparticles for plasmon-enhanced solar desalination. *Nat. Photonics* **10**, 393–398 (2016).

9. L. Zhou, Y. Tan, D. Ji, B. Zhu, P. Zhang, J. Xu, Q. Gan, Z. Yu, J. Zhu, Self-assembly of highly efficient, broadband plasmonic absorbers for solar steam generation. *Sci. Adv.* **2**, e1501227 (2016).

10. Z.-P. Yang, L. Ci, J. A. Bur, S.-Y. Lin, P. M. Ajayan, Experimental observation of an extremely dark material made by a low-density nanotube array. *Nano Lett.* **8**, 446–451 (2008).

11. Y. Salomon, N. Sternberg, I. Gouzman, G. Lempert, E. Grossman, D. Katsir, R. Cotostiano, T. Minton, Qualification of Acktar Black coatings for space application, paper presented at 11th International Symposium on Materials in a Space Environment, Aix-en-Provence, France, 15 to 18 September 2009, pp. 2–8; http://esmat.esa.int/materials_news/isme09/pdf/4-New/S7%20-%20Miron-Salomon.pdf.

12. C. Tu, W. Cai, X. Chen, X. Ouyang, H. Zhang, Z. Zhang, A 3D-structured sustainable solar-driven steam generator using super-black nylon flocking materials. *Small* **15**, 1902070 (2019).

13. J. Guo, D. Li, H. Zhao, W. Zou, Z. Yang, Z. Qian, S. Yang, M. Yang, N. Zhao, J. Xu, Cast-and-use super black coating based on polymer-derived hierarchical porous carbon spheres. *ACS Appl. Mater. Interfaces* **11**, 15945–15951 (2019).

14. S. Dou, H. Xu, J. Zhao, K. Zhang, N. Li, Y. Lin, L. Pan, Y. Li, Bioinspired microstructured materials for optical and thermal regulation. *Adv. Mater.* **33**, 2000697 (2021).

15. D. E. McCoy, T. Feo, T. A. Harvey, R. O. Prum, Structural absorption by barbule microstructures of super black bird of paradise feathers. *Nat. Commun.* **9**, 1 (2018).

16. A. L. Davis, K. N. Thomas, F. E. Goetz, B. H. Robison, S. Johnsen, K. J. Osborn, Ultra-black camouflage in deep-sea fishes. *Curr. Biol.* **30**, 3470–3476.e3 (2020).

17. A. L. Davis, H. F. Nijhout, S. Johnsen, Diverse nanostructures underlie thin ultra-black scales in butterflies. *Nat. Commun.* **11**, 1294 (2020).

18. H. Savin, P. Repo, G. von Gastrow, P. Ortega, E. Calle, M. Garín, R. Alcubilla, Black silicon solar cells with interdigitated back-contacts achieve 22.1% efficiency. *Nat. Nanotechnol.* **10**, 624–628 (2015).

19. K.-T. Lin, H. Lin, T. Yang, B. Jia, Structured graphene metamaterial selective absorbers for high efficiency and omnidirectional solar thermal energy conversion. *Nat. Commun.* **11**, 1389 (2020).

20. H. Ghasemi, G. Ni, A. M. Marconnet, J. Loomis, S. Yerci, N. Miljkovic, G. Chen, Solar steam generation by heat localization. *Nat. Commun.* **5**, 4449 (2014).

21. M. K. Hedayati, M. Javaherirahim, B. Mozooni, R. Abdelaziz, A. Tavassolizadeh, V. S. K. Chakravadhanula, V. Zaporojtchenko, T. Strunkus, F. Faupel, M. Elbahri, Design of a perfect black absorber at visible frequencies using plasmonic metamaterials. *Adv. Mater.* **23**, 5410–5414 (2011).

22. P. D. Dongare, A. Alabastri, S. Pedersen, K. R. Zodrow, N. J. Hogan, O. Neumann, J. Wu, T. Wang, A. Deshmukh, M. Elimelech, Q. Li, P. Nordlander, N. J. Halas, Nanophotonics-enabled solar membrane distillation for off-grid water purification. *Proc. Natl. Acad. Sci. U.S.A.* **114**, 6936–6941 (2017).

23. Y. Zhai, Y. Ma, S. N. David, D. Zhao, R. Lou, G. Tan, R. Yang, X. Yin, Scalable-manufactured randomized glass-polymer hybrid metamaterial for daytime radiative cooling. *Science* **355**, 1062–1066 (2017).

24. S. Zeng, S. Pian, M. Su, Z. Wang, M. Wu, X. Liu, M. Chen, Y. Xiang, J. Wu, M. Zhang, Q. Cen, Y. Tang, X. Zhou, Z. Huang, R. Wang, A. Tunuhe, X. Sun, Z. Xia, M. Tian, M. Chen, X. Ma, L. Yang, J. Zhou, H. Zhou, Q. Yang, X. Li, Y. Ma, G. Tao, Hierarchical-morphology metafabric for scalable passive daytime radiative cooling. *Science* **373**, 692–696 (2021).

25. L. Zhu, A. P. Raman, S. Fan, Radiative cooling of solar absorbers using a visibly transparent photonic crystal thermal blackbody. *Proc. Natl. Acad. Sci. U.S.A.* **112**, 12282–12287 (2015).

26. H. Zhang, K. C. S. Ly, X. Liu, Z. Chen, M. Yan, Z. Wu, X. Wang, Y. Zheng, H. Zhou, T. Fan, Biologically inspired flexible photonic films for efficient passive radiative cooling. *Proc. Natl. Acad. Sci. U.S.A.* **117**, 14657–14666 (2020).

27. C. Yu, Y. Li, X. Zhang, X. Huang, V. Malyarchuk, S. Wang, Y. Shi, L. Gao, Y. Su, Y. Zhang, H. Xu, R. T. Hanlon, Y. Huang, J. A. Rogers, Adaptive optoelectronic camouflage systems with designs inspired by cephalopod skins. *Proc. Natl. Acad. Sci. U.S.A.* **111**, 12998–13003 (2014).

28. K. Tang, K. Dong, J. Li, M. P. Gordon, F. G. Reichertz, H. Kim, Y. Rho, Q. Wang, C.-Y. Lin, C. P. Grigoropoulos, A. Javey, J. J. Urban, J. Yao, R. Levinson, J. Wu, Temperature-adaptive radiative coating for all-season household thermal regulation. *Science* **374**, 1504–1509 (2021).

29. X. Ao, B. Li, B. Zhao, M. Hu, H. Ren, H. Yang, J. Liu, J. Cao, J. Feng, Y. Yang, Z. Qi, L. Li, C. Zou, G. Pei, Self-adaptive integration of photothermal and radiative cooling for continuous energy harvesting from the sun and outer space. *Proc. Natl. Acad. Sci. U.S.A.* **119**, e2120557119 (2022).

30. K. Amemiya, H. Koshikawa, M. Imbe, T. Yamaki, H. Shitomi, Perfect blackbody sheets from nano-precision microtextured elastomers for light and thermal radiation management. *J. Mater. Chem. C* **7**, 5418–5425 (2019).

31. J. Lehman, C. Yung, N. Tomlin, D. Conklin, M. Stephens, Carbon nanotube-based black coatings. *Appl. Phys. Rev.* **5**, 011103 (2018).

32. C. S. Yung, N. A. Tomlin, K. Heuerman, M. W. Keller, M. G. White, M. Stephens, J. H. Lehman, Plasma modification of vertically aligned carbon nanotubes: Superhydrophobic surfaces with ultra-low reflectance. *Carbon* **127**, 195–201 (2018).

33. T. Yanagishita, K. Yasui, T. Kondo, Y. Kawamoto, K. Nishio, H. Masuda, Antireflection polymer surface using anodic porous alumina molds with tapered holes. *Chem. Lett.* **36**, 530–531 (2007).

34. Z. Diao, M. Kraus, R. Brunner, J.-H. Dirks, J. P. Spatz, Nanostructured stealth surfaces for visible and near-infrared light. *Nano Lett.* **16**, 6610–6616 (2016).

35. G. Tan, J.-H. Lee, Y.-H. Lan, M.-K. Wei, L.-H. Peng, I.-C. Cheng, S.-T. Wu, Broadband antireflection film with moth-eye-like structure for flexible display applications. *Optica* **4**, 678–683 (2017).

36. N. Liu, M. Mesch, T. Weiss, M. Hentschel, H. Giessen, Infrared perfect absorber and its application as plasmonic sensor. *Nano Lett.* **10**, 2342–2348 (2010).

37. A. Moreau, C. Ciracì, J. J. Mock, R. T. Hill, Q. Wang, B. J. Wiley, A. Chilkoti, D. R. Smith, Controlled-reflectance surfaces with film-coupled colloidal nanoantennas. *Nature* **492**, 86–89 (2012).

38. Y. Shimizu, H. Koshikawa, M. Imbe, T. Yamaki, K. Amemiya, Large-area perfect blackbody sheets having aperiodic array of surface micro-cavities for high-precision thermal imager calibration. *Opt. Express* **28**, 22606–22616 (2020).

39. Y. Shimizu, H. Koshikawa, M. Imbe, T. Yamaki, K. Godo, N. Sasajima, K. Amemiya, Micro-cavity perfect blackbody composite with good heat transfer towards a flat-plate reference radiation source for thermal imagers. *Opt. Lett.* **46**, 4871–4874 (2021).

40. Y. Shimizu, M. Imbe, K. Godo, N. Sasajima, H. Koshikawa, T. Yamaki, K. Amemiya, High-precision flat-plate reference infrared radiator using perfect blackbody composite with a microcavity structure. *Appl. Optics* **61**, 517–522 (2022).

41. D. Balgude, A. S. Sabnis, CNSL: An environment friendly alternative for the modern coating industry. *J. Coatings Technol. Res.* **11**, 169–183 (2014).

42. S. Kobayashi, H. Uyama, R. Ikeda, Artificial Urushi. *Chemistry* **7**, 4754–4760 (2001).

43. K. Mizuno, J. Ishii, H. Kishida, Y. Hayamizu, S. Yasuda, D. N. Futaba, M. Yumura, K. Hata, A black body absorber from vertically aligned single-walled carbon nanotubes. *Proc. Natl. Acad. Sci. U.S.A.* **106**, 6044–6047 (2009).

44. A. Deinega, I. Valuev, B. Potapkin, Y. Lozovik, Minimizing light reflection from dielectric textured surfaces. *J. Opt. Soc. Am. A* **28**, 770–777 (2011).

45. K. Amemiya, H. Koshikawa, T. Yamaki, Y. Maekawa, H. Shitomi, T. Numata, K. Kinoshita, M. Tanabe, D. Fukuda, Fabrication of hard-coated optical absorbers with microstructured surfaces using etched ion tracks: Toward broadband ultra-low reflectance. *Nucl. Instrum. Methods Phys. Res. B.* **356–357**, 154–159 (2015).

46. P. Apel, Swift ion effects in polymers: Industrial applications. *Nucl. Instrum. Methods Phys. Res. B.* **208**, 11–20 (2003).

47. M. Fromm, S. Kodaira, T. Kusumoto, R. Barillon, T. Yamauchi, Role of intermediate species in the formation of ion tracks in PADC: A review. *Polym. Degrad. Stab.* **161**, 213–224 (2019).

48. N. Yasuda, M. Yamamoto, K. Amemiya, H. Takahashi, A. Kyan, K. Ogura, Track sensitivity and the surface roughness measurements of CR-39 with atomic force microscope. *Radiat. Meas.* **31**, 203–208 (1999).

49. B. G. Cartwright, E. K. Shirk, P. B. Price, A nuclear-track-recording polymer of unique sensitivity and resolution. *Nucl. Instrum. Methods* **153**, 457–460 (1978).

50. V. Ghai, H. Singh, P. K. Agnihotri, Near perfect thin film flexible broadband optical absorber with high thermal/electrical conductivity. *J. Appl. Polym. Sci.* **137**, 48855 (2020).

51. H. Watanabe, A. Fujimoto, A. Takahara, Characterization of catechol-containing natural thermosetting polymer "urushiol" thin film. *J. Polym. Sci. Part A Polym. Chem.* **51**, 3688–3692 (2013).

52. X. Fan, W. Zheng, D. J. Singh, Light scattering and surface plasmons on small spherical particles. *Light Sci. Appl.* **3**, e179 (2014).

53. S.-P. Rwei, F.-H. Ku, K.-C. Cheng, Dispersion of carbon black in a continuous phase: Electrical, rheological, and morphological studies. *Colloid Polym. Sci.* **280**, 1110–1115 (2002).

54. T. H. Fung, T. Veeken, D. Payne, B. Veettil, A. Polman, M. Abbott, Application and validity of the effective medium approximation to the optical properties of nano-textured silicon coated with a dielectric layer. *Opt. Express* **27**, 38645–38660 (2019).

55. W. Sun, A. Du, Y. Feng, J. Shen, S. Huang, J. Tang, B. Zhou, Super black material from low-density carbon aerogels with subwavelength structures. *ACS Nano* **10**, 9123–9128 (2016).

56. J. Guo, D. Li, Z. Qian, H. Luo, M. Yang, Q. Wang, J. Xu, N. Zhao, Carbon vesicles: A symmetry-breaking strategy for wide-band and solvent-processable ultrablack coating materials. *Adv. Funct. Mater.* **30**, 1909877 (2020).

57. H. Berneth, Azine dyes, in *Ullmann's Encyclopedia of Industrial Chemistry* (Wiley-VCH Verlag GmbH & Co. KGaA, 2008).

58. M. A. Eddings, M. A. Johnson, B. K. Gale, Determining the optimal PDMS-PDMS bonding technique for microfluidic devices. *J. Micromech. Microeng.* **18**, 067001 (2008).

59. S. Kurashima, T. Satoh, Y. Saitoh, W. Yokota, Irradiation facilities of the Takasaki Advanced Radiation Research Institute. *Quantum Beam Sci.* **1**, 2 (2017).

60. S. Kodaira, N. Yasuda, T. Konishi, H. Kitamura, M. Kurano, H. Kawashima, Y. Uchihori, K. Ogura, E. R. Benton, Calibration of CR-39 with atomic force microscope for the measurement of short range tracks from proton-induced target fragmentation reactions. *Radiat. Meas.* **50**, 232–236 (2013).

61. CIE, *CIE 015:2018 Colorimetry, 4th Edition* (CIE, 2018); doi:10.25039/TR.015.2018.

62. M. Tanabe, K. Amemiya, T. Numata, D. Fukuda, Spectral supralinearity of silicon photodiodes in visible light due to surface recombination. *Appl. Optics* **55**, 3084–3089 (2016).

63. M. Tanabe, K. Kinoshita, Supralinear behavior and its wavelength dependence of silicon photodiodes with over-filled illumination in visible range. *Appl. Optics* **57**, 3575–3580 (2018).

64. A. Milionis, E. Loth, I. S. Bayer, Recent advances in the mechanical durability of superhydrophobic materials. *Adv. Colloid Interface Sci.* **229**, 57–79 (2016).

65. P. Laven, Simulation of rainbows, coronas, and glories by use of Mie theory. *Appl. Optics* **42**, 436–444 (2003).

66. I. Sadeghi, A. Munoz, P. Laven, W. Jarosz, F. Seron, D. Gutierrez, H. W. Jensen, Physically-based simulation of rainbows. *ACM Trans. Graph.* **31**, 1–12 (2012).

67. H. Chang, T. T. Charalampopoulos, Determination of the wavelength dependence of refractive indices of flame soot. *Proc. R. Soc. A Math. Phys. Eng. Sci.* **430**, 577–591 (1990).

68. E. D. Palik, G. Ghosh, *Handbook of Optical Constants of Solids* (Academic Press, 1998), vol. 3.

69. J. B. Yadav, R. B. Patil, R. K. Puri, V. Puri, Studies on spin coated PANI/PMMA composite thin film: Effect of post-deposition heating. *Appl. Surf. Sci.* **255**, 2825–2829 (2008).

70. S. A. Prahl, M. J. C. van Gemert, A. J. Welch, Determining the optical properties of turbid media by using the adding-doubling method. *Appl. Optics* **32**, 559–568 (1993).

71. A. F. Oskooi, D. Roundy, M. Ibanescu, P. Bermel, J. D. Joannopoulos, S. G. Johnson, Meep: A flexible free-software package for electromagnetic simulations by the FDTD method. *Comput. Phys. Commun.* **181**, 687–702 (2010).

72. M. R. Querry, *Optical Constants of Minerals and Other Materials from the Millimeter to the Ultraviolet* (Chemical Research, Development & Engineering Center, U.S. Army Armament Munitions Chemical Command, 1987).
